# Sorafenib plus hepatic arterial infusion chemotherapy versus sorafenib plus transarterial chemoembolization in advanced hepatocellular carcinoma: a phase III trial and biomarker analyses

**DOI:** 10.1016/j.jncc.2026.04.002

**Published:** 2026-05-19

**Authors:** Zhicheng Lai, Zefeng Du, Lichang Huang, Qijiong Li, Yexing Huang, Li Xu, Wei Wei, Yaojun Zhang, Minshan Chen, Ming Shi, Anna Kan, Minke He

**Affiliations:** a Department of Hepatobiliary Oncology, State Key Laboratory of Oncology in South China, Guangdong Provincial Clinical Research Center for Cancer, Sun Yat-sen University Cancer Center, Guangzhou, China; b State Key Laboratory of Oncology in South China; Guangdong Provincial Clinical Research Center for Cancer, Sun Yat-sen University Cancer Center, Guangzhou, China

**Keywords:** Hepatocellular carcinoma, Sorafenib, Transcatheter arterial chemoembolization, Hepatic arterial infusion chemotherapy, Phosphofructokinase

## Abstract

**Objective:**

Although sorafenib plus transcatheter arterial chemoembolization (SoraTACE) has been widely applied for advanced hepatocellular carcinoma (HCC) in most Asian countries, sorafenib plus hepatic arterial infusion chemotherapy (SoraHAIC) may be a better alternative. This phase III trial assessed the efficacy and safety of SoraHAIC versus SoraTACE in patients with advanced HCC.

**Methods:**

Participants were randomly assigned (2:1) to receive SoraHAIC (400 mg of sorafenib twice daily plus FOLFOX-HAIC every 3 weeks) or SoraTACE (400 mg of sorafenib plus TACE). The primary endpoint was overall survival (OS).

**Results:**

From August 2016 to October 2020, 207 participants were allocated to receive SoraHAIC (*n =* 141) or SoraTACE (*n* = 66). SoraHAIC significantly improved OS (median OS, 15.7 vs. 11.2 months; hazard ratio [HR], 0.58 [95% CI: 0.42, 0.80]; *P <* 0.001) and progression-free survival (median, 7.2 vs. 4.1 months; HR, 0.48 [95% CI: 0.35, 0.65]; *P <* 0.001) compared to SoraTACE. The incidence of grade 3-4 treatment-related adverse events was significantly lower with SoraHAIC (39.3%) than SoraTACE (56.7%) (*P <* 0.023). Biomarker exploration indicated that patients with higher phosphofructokinase (PFKM) expression benefitted more from SoraTACE.

**Conclusions:**

SoraHAIC significantly improves OS over SoraTACE in participants with advanced HCC. Participants with high PFKM expression might benefit more from SoraTACE.

## 1 Introduction

Approximately 10%–40% of hepatocellular carcinoma (HCC) patients are diagnosed at an advanced stage during their initial presentation, with this proportion reported to be about 54.9% in China.^1^^,^^2^ Sorafenib is one of the standards of care for patients with advanced disease,^3^ and confers a 2.8-month survival advantage compared with placebo.^4^ Moreover, compared with the overall survival (OS) of HCC patients receiving sorafenib monotherapy in Western countries (median OS, 10.7 months), that in Asian patients has not improved substantially with sorafenib monotherapy (median OS, 6.5–7.1 months).^5^ The possible reason may be that patients in Asia commonly present to the clinic with large tumors, indicating poor reagent penetration of the tumor.^6^ Therefore, combining locoregional therapies with sorafenib to reduce the intrahepatic tumor burden is suggested in Asia.^7^

Transcatheter arterial chemoembolization (TACE) has been adapted as locoregional therapy for advanced HCC,^1^ and selected Barcelona Clinic Liver Cancer (BCLC) stage C patients indeed benefit from TACE monotherapy.^8^, ^9^, ^10^, ^11^ It was therefore assumed that the combination of sorafenib and TACE (SoraTACE) could synergistically enhance treatment efficacy.^12^ Although the phase III STAH study did not demonstrate a significant improvement in median OS with SoraTACE compared with sorafenib monotherapy, this combination therapy significantly prolonged progression-free survival (PFS).^13^ Several retrospective studies and systematic review had also suggested the efficacy and safety of SoraTACE.^14^, ^15^, ^16^ Although TACE is not endorsed by the practice guidelines of major Western scientific associations such as European Association for the Study of the Liver (EASL), European Society for Medical Oncology (ESMO), American Society of Clinical Oncology (ASCO), or American Association for the Study of Liver Diseases (AASLD), TACE has been listed as the recommended treatment for advanced HCC in most Asian countries.^7^^,^^17^, ^18^, ^19^ Recently, an overview of advances in the treatment of HCC also recommended TACE for advanced HCC.^20^

We previously reported that increasing the dosage of chemotherapeutics for chemolipiodolization can improve the disease prognosis.^21^ In our published series of studies, hepatic arterial infusion chemotherapy (HAIC) has demonstrated significant antitumor efficacy in the intermediate-advanced stage of HCC.^22^, ^23^, ^24^, ^25^ Notably, for patients with large, unresectable HCCs, HAIC resulted in significantly improved clinical outcomes compared with those of TACE, and this was attributed to sustained high-concentration drug exposure.^26^^,^^27^ In this study, we aimed to evaluate the synergistic efficacy of SoraHAIC and determine whether this combination would be beneficial in the treatment of advanced HCC patients with large tumor burdens in the Chinese population, in contrast to SoraTACE, which was not beneficial in this way. Additionally, we performed a comprehensive biomarker study to address the shortcomings of current tests for predicting treatment response.

## 2 Materials and methods

### 2.1 Study design and participants

Eligible participants were 18 years of age or older and had BCLC stage C HCC, with the diagnosis confirmed by histologic or cytologic analysis or clinical features according to the EASL clinical practice guidelines.^28^ Eligible participants had not previously received treatment or had at least one measurable lesion, as defined by the EASL criteria; did not have cirrhosis or had a cirrhotic status of Child‒Pugh class A; and had adequate hematologic and organ function. Among the key exclusion criteria were main trunk portal vein occlusion; history of human immunodeficiency virus (HIV) infection, organ allograft, and central nervous system tumors; evidence of hepatic decompensation; bleeding diathesis or events; and allergy to the investigational agents or any agent given in association with this trial. The full eligibility and exclusion criteria are provided in the Supplementary Materials.

Participants were randomly assigned in a 2:1 ratio to receive SoraHAIC or SoraTACE. Randomization was performed centrally via a computer-generated sequence and was stratified according to tumor size (≤ 10 cm vs. > 10 cm).

### 2.2 Procedure

The participants assigned to the SoraTACE group received 400 mg of sorafenib orally twice daily, and TACE was performed according to our previously reported protocol.^21^ A 3.5 French catheter was inserted into the celiac trunk or superior mesenteric artery for arteriography. A 2.7 French microcatheter was then superselectively placed into the feeding arteries of the tumors. Chemolipiodolization was performed with 50 mg of epirubicin and 50 mg of lobaplatin mixed with Lipiodol. Subsequently, embolization was performed via the injection of polyvinyl alcohol particles. Repeated TACE cycles were performed every 6 weeks. The participants assigned to the SoraHAIC group received sorafenib in the same way, and HAIC treatment was divided into 3-week cycles. The microcatheter was advanced into the hepatic artery according to our previously reported protocol on day 1 in every cycle of treatment.^24^ The participants were transferred to the inpatient ward for drug infusion via the hepatic artery as follows: oxaliplatin, 85 mg/m² from hour 0 to 2 on day 1; leucovorin, 400 mg/m² from hour 2 to 3 on day 1; and fluorouracil, 400 mg/m² bolus at hour 3 and 2400 mg/m² over 24 h. After HAIC was completed, the catheter and sheath were removed. No implanted port catheter system was used, and repeated femoral artery puncture and catheterization were performed in each HAIC cycle. HAIC was repeated every 3 weeks for up to six cycles. Patients with hepatitis B virus (HBV) infection in this study concomitantly received effective antiviral therapy. The criteria for treatment crossover, dose reduction, delay, discontinuation, and poststudy treatment are shown in the Supplementary Materials.

### 2.3 Outcomes

The primary endpoint was OS (the time from randomization to death from any cause or the date of the last follow-up if the patient was alive). The secondary endpoints were PFS (the interval from randomization to disease progression according to the RECIST v1.1 or death from any cause, whichever occurred first), tumor response (assessed by investigators using the RECIST v1.1), and adverse events (evaluated by vital signs and clinical laboratory test results and assessment of the incidence and severity of adverse events according to the National Cancer Institute [NCI] Common Terminology Criteria for Adverse Events, version 4.0).

### 2.4 Biomarker screening

Our goal was to identify biomarkers that have the potential for clinical application. To this end, we examined data from two cohorts. Briefly, we first conducted RNA sequencing (RNA-seq) on 80 samples derived from our previous phase III trial (FOLFOX-HAIC, *n* = 46; TACE, *n* = 34; NCT02973685) as the discovery cohort and 93 samples derived from this study (SoraHAIC, *n* = 50; SoraTACE, *n* = 43) as the validation cohort. The samples were then divided into four groups according to their therapy regimens and outcomes. The detailed methods are provided in the Supplementary Materials.

### 2.5 Statistical analysis

We used SPSS (version 25.0) and RStudio (R version 4.3.0) for all analyses. The sample size was based on the assumption that the median OS of patients receiving SoraTACE or SoraHAIC would be 10 months and 16 months, respectively. To detect this difference with 80% power and a two-sided α of 0.05, we calculated that the required number of events would be observed if 192 patients were enrolled, with an enrollment period of 36 months and a follow-up period of 18 months. On the basis of an estimated dropout rate of 7%, target enrollment was set at 206 (69 patients assigned to SoraTACE and 137 patients assigned to SoraHAIC). Detailed statistical descriptions and analytical methods have been provided in the Supplementary Materials.

## 3 Results

### 3.1 Patient

**Fig. 1:**
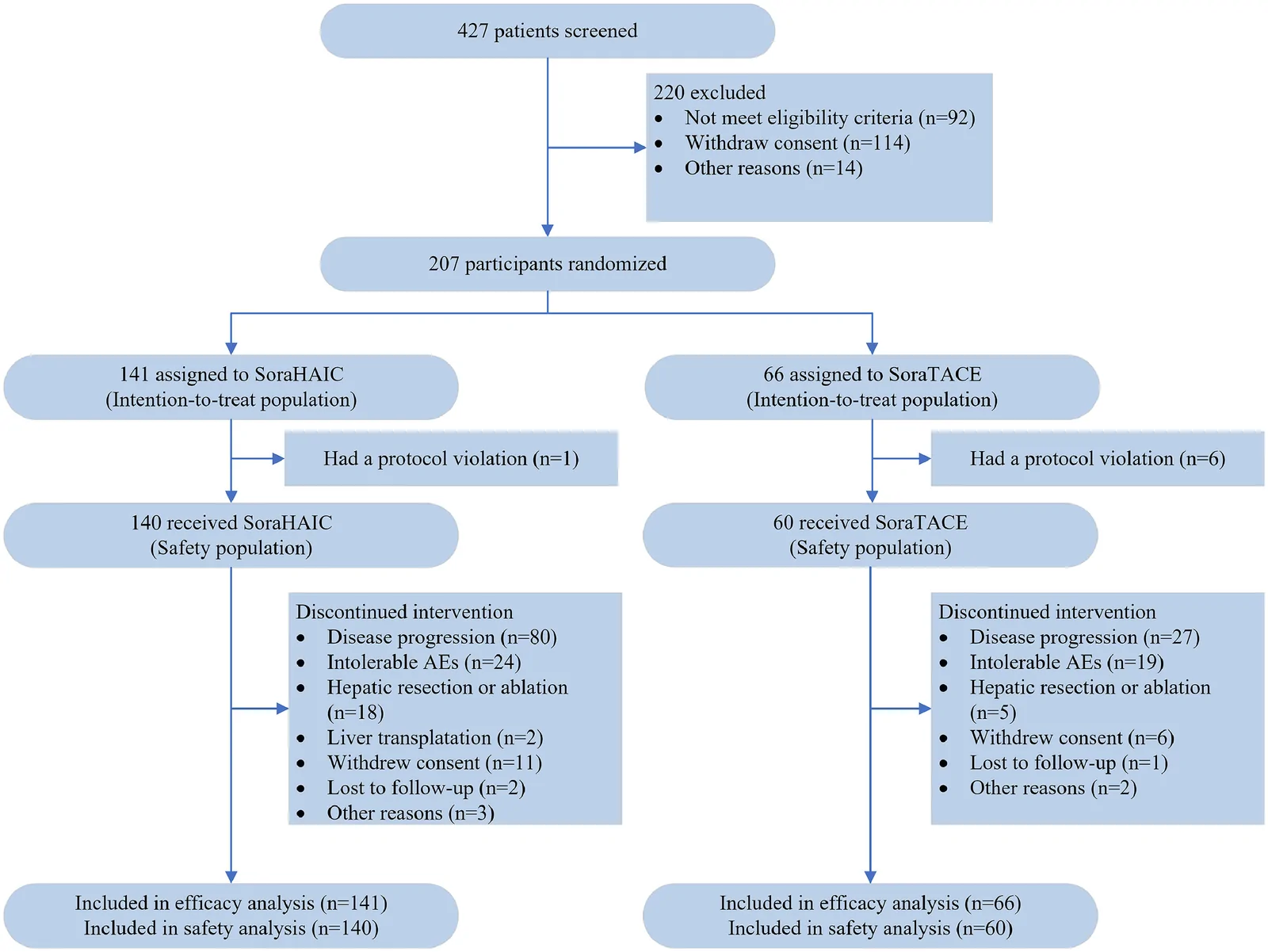
Trial flowchart. AE, adverse event; HAIC, hepatic arterial infusion chemotherapy; Sora, sorafenib; TACE, transarterial chemoembolization.

From August 4, 2016, to October 25, 2020, a total of 427 patients from Sun Yat-sen University Cancer Center were screened to determine their eligibility for the trial, and 220 patients were excluded (Fig. 1). In total, 207 participants were randomized and assigned to receive SoraHAIC treatment (*n =* 141) or SoraTACE treatment (*n =* 66). As of the data cutoff date (May 1, 2022), the median follow-up duration was 39.6 months (Interquartile range [IQR]: 28.8, 53.4). The median age was 50 years (IQR: 40, 56), and 184 participants (88.9%) were male. Most participants had HBV infection as an etiology of HCC (*n =* 196, 94.7%). In the SoraHAIC group, 47.5% of participants had a maximum tumor diameter ≤ 10 cm and 52.5% had a diameter > 10 cm, whereas the corresponding proportions in the SoraTACE group were 53% and 47% (*P =* 0.46). The proportion of participants with multinodular disease was 84.4% and 81.8%, respectively (*P =* 0.64). The proportion of participants with portal vein tumor thrombus (PVTT, Vp0–Vp4) (*P =* 0.11) or extrahepatic spread (*P =* 0.10) was similar between the two groups, indicating a comparable degree of vascular involvement and metastatic burden (Table 1). Treatment exposure differed markedly between cohorts: the SoraHAIC group underwent a median of 4 HAIC cycles, while the SoraTACE group received a median of 1 TACE procedure. Similarly, sorafenib was administered for a longer duration in the SoraHAIC group (median 4.8 vs. 3.4 months; *P =* 0.01). Thirteen participants from the SoraTACE group crossed over to SoraHAIC treatment. The differences in subsequent therapies between the two groups were small and not considered clinically meaningful (Supplementary Table 1).

**Table 1:** Baseline characteristics.

Subsets	SoraHAIC (*n* = 141)	SoraTACE (*n* = 66)	*P*
Age, median (IQR), years	50 (39-56)	50.5 (43-58)	0.250
Sex, No. (%)			0.430
Male	127 (90.1)	57 (86.4)	
Female	14 (9.9)	9 (13.6)	
ECOG score, No. (%)			0.160
0	34 (24.1)	22 (33.3)	
1-2	107 (75.9)	44 (66.7)	
HBV infection, No. (%)			0.500
Negative	9 (6.4)	2 (3)	
Positive	132 (93.6)	64 (97)	
Cirrhosis, No. (%)			0.410
None or mild	46 (69.7)	106 (75.2)	
Moderate or severe	35 (24.8)	20 (30.3)	
Portal hypertension, No. (%)			1.000
No	61 (92.4)	131 (92.9)	
Yes	5 (7.6)	10 (7.1)	
Neutrophil: lymphocyte ratio, No. (%)			0.960
≤ 3	71 (50.4)	33 50)	
> 3	70 (49.6)	33 (50)	
ALT, median (IQR), U/L	49 (31.6-70.0)	46.5 (33.8-75.2)	0.900
AST, median (IQR), U/L	65.2 (47.2-115.4)	72.6 (45.6-99.4)	0.830
TBIL, median (IQR), µmol/L	16.3 (11.9-20.3)	15.6 (11.4-19.4)	0.440
ALB, median (IQR), g/dL	39.9 (37.3-43.4)	41.2 (37.0-44.1)	0.310
PT, median (IQR), seconds	12.4 (11.8-13.0)	12.5 (11.6-13.1)	0.860
AFP, median (IQR), ng/mL	9672 (326.3-104,681.5)	4684.5 (94.3-74,401.3)	0.280
Maximum tumor diameter, No. (%), cm			0.460
≤ 10	67 (47.5)	35 (53)	
> 10	74 (52.5)	31 (47)	
Tumor number, No. (%)			0.640
Single	22 (15.6)	12 (18.2)	
Multiple	119 (84.4)	54 (81.8)	
PVTT, No. (%)			0.110
Vp0	25 (17.7)	18 (27.3)	
Vp1-2	45 (31.9)	26 (39.4)	
Vp3	65 (46.1)	21 (31.8)	
Vp4	6 (4.3)	1 (1.5)	
Extrahepatic spread, No. (%)			0.100
Absent	109 (77.3)	44 (66.7)	
Present	32 (22.7)	22 (33.3)	
Crossover treatments, No.	0	13	<0.001
Subsequent therapies, No. (%)			
HAIC	20 (14.2)	17 (25.8)	0.043
TACE	11 (7.8)	13 (19.7)	0.013
Resection	15 (10.6)	2 (3.0)	0.063
Other TKI	32 (22.7)	10 (15.2)	0.210
PD-1 antibody	20 (14.2)	5 (7.6)	0.170

Abbreviations: AFP, alpha-fetoprotein; ALT, alanine aminotransferase; AST, aspartate aminotransferase; ECOG, eastern cooperative oncology group; HAIC, hepatic arterial infusion chemotherapy; HBV, hepatitis B virus; IQR, interquartile range; PT, prothrombin time; PVTT, portal vein tumor thrombus; TACE, transarterial chemoembolization; TBIL, total bilirubin; TKI, tyrosine kinase inhibitor.

### 3.2 Efficacy

**Fig. 2:**
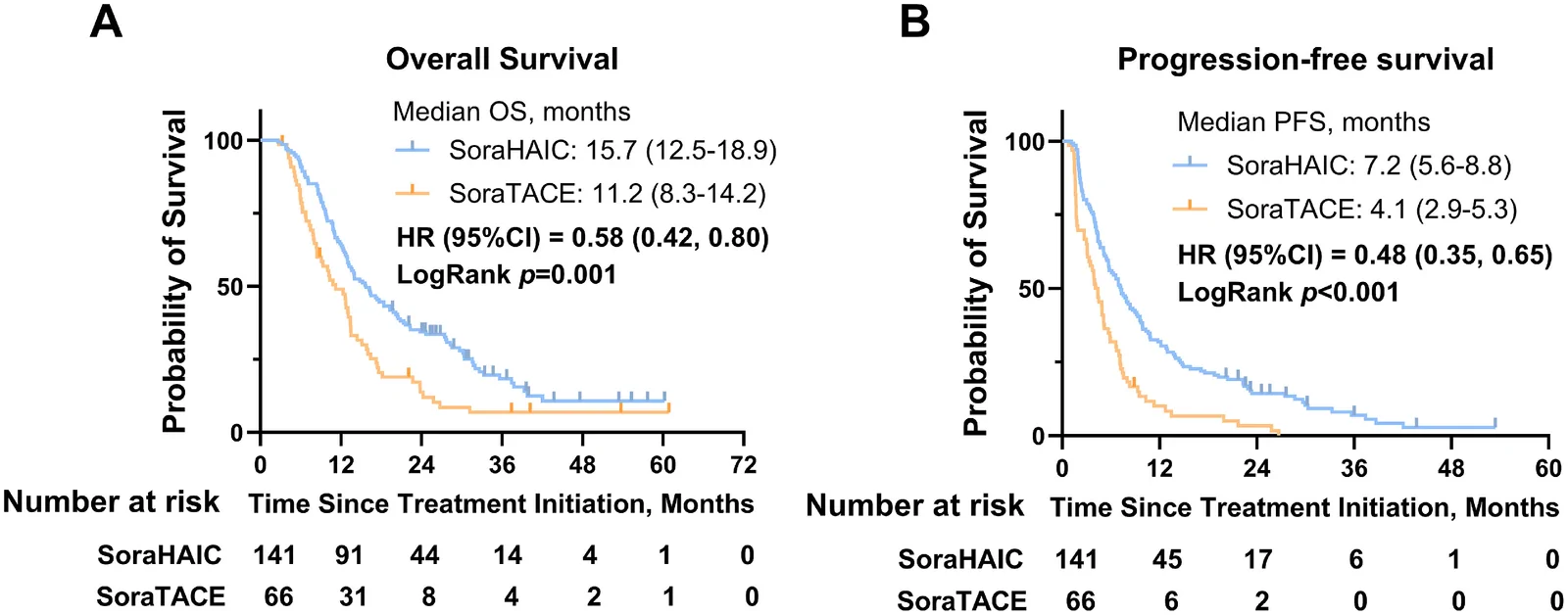
Survival analysis. (A, B) Kaplan‒Meier curves of overall survival (A) and progression-free survival (B). CI, confidence interval; HAIC, hepatic arterial infusion chemotherapy; HR, hazard ratio; OS, overall survival; PFS, progression-free survival; Sora, sorafenib; TACE, transarterial chemoembolization.

As of the specified data cutoff date, the median OS was 15.7 months (95% confidence interval [CI]: 12.5, 18.9) in the SoraHAIC group and 11.2 months (95% CI: 8.3, 14.2) in the SoraTACE group (hazard ratio [HR], 0.58 [95% CI: 0.42, 0.80]; *P =* 0.001) (Fig. 2A). Multivariate analysis demonstrated that the treatment group, Eastern Cooperative Oncology Group performance status (ECOG PS) score, cirrhosis status, and alpha-fetoprotein (AFP) levels were independent risk factors for OS (Supplementary Table 2).

The median PFS was 7.2 months (95% CI: 5.6, 8.8) in the SoraHAIC group and 4.1 months (95% CI: 2.9, 5.3) in the SoraTACE group (HR, 0.48 [95% CI: 0.35, 0.65]; *P <* 0.001) (Fig. 2B). Treatment group and extrahepatic spread were found to be independent risk factors for PFS (Supplementary Table 2). Subgroup analysis revealed that patients derived consistent clinical benefit from SoraHAIC therapy regardless of tumor size (≤ 10 cm vs. > 10 cm) (Supplementary Fig. 1A and B).

The investigator-assessed tumor objective response rates (ORR) per RECIST v1.1 were 46.8% (95% CI: 38.4%, 55.4%) with SoraHAIC and 10.6% (95% CI: 4.4%, 20.6%) with SoraTACE (*P <* 0.001), and the modified RECIST (mRECIST) rates were 52.5% (95% CI: 43.9%, 60.9%) and 30.3% (95% CI: 19.6%, 42.9%), respectively (*P =* 0.003) (Table 2). In total, 10 participants (7.1%) in the SoraHAIC group, as compared with 3 participants (4.5%) in the SoraTACE group, had a complete response according to mRECIST (Supplementary Table 3). The disease control rate was 73.8% with SoraHAIC and 59.1% with SoraTACE (*P =* 0.033).

**Table 2:** Tumor response.*

	Overall						Intrahepatic					
Pathological response, No. (%)	RECIST 1.1		*P*	mRECIST		*P*	RECIST 1.1		*P*	mRECIST		*P*
	SoraHAIC (*n* = 141)	SoraTACE (*n* = 66)		SoraHAIC (*n* = 141)	SoraTACE (*n* = 66)		SoraHAIC (*n* = 141)	SoraTACE (*n* = 66)		SoraHAIC (*n* = 141)	SoraTACE (*n* = 66)	
CR	0	0	-	10 (7.1)	3 (4.5)	0.690	0	0	-	12 (8.5)	3 (4.5)	0.460
PR	66 (46.8)	7 (10.6)	< 0.001	64 (45.4)	17 (25.8)	0.007	69 (48.9)	7 (10.6)	<0.001	78 (55.3)	22 (33.3)	0.003
SD	38 (27)	32 (48.5)	0.002	30 (21.3)	19 (28.8)	0.240	40 (28.4)	36 (54.5)	<0.001	20 (14.2)	23 (34.8)	0.001
PD	29 (20.6)	23 (34.8)	0.027	29 (20.6)	23 (34.8)	0.027	24 (17)	19 (28.8)	0.520	23 (16.3)	14 (21.2)	0.390
NA	8 (5.7)	4 (6.1)	1.000	8 (5.7)	4 (6.1)	1.000	8 (5.7)	4 (6.1)	1.000	8 (5.7)	4 (6.1)	1.000
ORR	66 (46.8)	7 (10.6)	< 0.001	74 (52.5)	20 (30.3)	0.003	69 (48.9)	7 (10.6)	< 0.001	90 (63.8)	25 (37.9)	< 0.001
DCR	104 (73.8)	39 (59.1)	0.033	104 (73.8)	39 (59.1)	0.033	109 (77.3)	43 (62.1)	0.065	110 (78)	48 (72.7)	0.400

⁎ The frequency of radiological assessments in our study is every six weeks. Abbreviations: CR, complete response; DCR, disease control rate; mRECIST, modified response evaluation criteria in solid tumors; NA, not available; ORR, objective response rate; PD, progressive disease; PR, partial response; RECIST, response evaluation criteria in solid tumors; SD, stable disease.

### 3.3 Safety

The profiles of adverse events (AEs) that occurred in ≥ 10% of the overall study participants are presented in Table 3. The overall incidence of fever, alanine aminotransferase (ALT) elevation, aspartate aminotransferase (AST) elevation, and total bilirubin (TBIL) elevation were significantly greater in the SoraTACE group than in the SoraHAIC group, whereas hypoalbuminemia was more common in the SoraHAIC group. The incidence of palmar-plantar erythrodysesthesia syndrome was comparable between the two groups. Additionally, 3 participants in the SoraHAIC group and 4 participants in the SoraTACE group experienced upper gastrointestinal bleeding. Grade 3–4 adverse events were noted in 89 participants (44.5%), with the most frequent event being increased AST (*n =* 50, 25.0%), the incidence of which was significantly higher in the SoraTACE group (23 [16.4%] vs. 27 [45.0%]; *P <* 0.001). Serious AEs occurred more frequently with SoraTACE (16 participants [26.7%]) than with the SoraHAIC group (22 participants [15.7%]) (*P =* 0.070). No participant experienced treatment-related death.

**Table 3:** Treatment-related adverse events.

Adverse effects, No. (%)	SoraHAIC (*n* = 140)				SoraTACE (*n* = 60)				*P*	*P* for G3-G4
	Any grade	Grade 1-2	Grade 3	Grade 4	Any grade	Grade 1-2	Grade 3	Grade 4		
Hypertension	39 (28.9)	34 (24.3)	5 (3.6)	0	21 (35.0)	18 (30.0)	3 (5.0)	0	0.790	0.940
Fatigue	60 (42.9)	50 (35.7)	10 (7.1)	0	28 (46.7)	25(41.6)	3 (5.0)	0	0.840	0.800
Fever	20 (14.3)	18 (12.9)	2 (1.4)	0	25 (41.7)	23 (38.3)	2 (3.3)	0	< 0.001	0.590
Sensory neuropathy	25 (17.9)	24 (17.2)	1 (0.7)	0	3 (5.0)	3 (5.0)	0	0	0.092	1.000
Abdominal pain	54 (38.6)	51 (36.4)	3 (2.1)	0	26 (43.3)	25 (41.7)	1 (1.7)	0	0.500	1.000
Nausea	84 (60.0)	78 (55.7)	6 (4.3)	0	32 (53.3)	31 (51.7)	1 (1.7)	0	0.730	0.680
Vomit	68 (48.6)	59 (42.2)	7 (5.0)	2 (1.4)	24 (40.0)	21 (35.0)	3 (5.0)	0	0.410	0.950
Diarrhea	40 (28.6)	33 (33.5)	7 (5.0)	0	16 (26.7)	14 (23.4)	2 (3.3)	0	0.990	0.880
Hand-foot syndrome	50 (35.7)	40 (28.6)	10 (7.1)	0	22 (36.7)	17 (28.3)	5 (8.3)	0	0.970	1.000
Neutropenia	27 (19.3)	19 (13.6)	6 (4.3)	2 (1.4)	8 (13.3)	7 (11.7)	1 (1.7)	0	0.610	0.370
Anemia	86 (61.4)	84 (60.0)	2 (1.4)	0	30 (50.0)	30 (50.0)	0	0	0.290	1.000
Thrombocytopenia	69 (49.3)	59 (42.1)	8 (5.7)	2 (1.4)	22 (36.7)	18 (30.0)	4 (6.7)	0	0.390	1.000
Elevated ALT	93 (66.4)	86 (61.4)	7 (5.0)	0	52 (86.7)	39 (65.0)	11 (18.3)	2 (3.3)	<0.001	<0.001
Elevated AST	133 (95)	110 (78.6)	22 (15.7)	1 (0.7)	59 (98.3)	32 (53.3)	22 (36.7)	5 (8.3)	<0.001	<0.001
Hyperbilirubinemia	64 (45.7)	62 (44.3)	2 (1.4)	0	39 (65.0)	36 (60.0)	3 (5.0)	0	0.036	0.160
Hypoalbuminemia	136 (97.1)	136 (97.1)	0	0	52 (86.7)	52 (86.7)	0	0	0.008	-
Grade 3-4	55 (39.3)				34 (56.7)				0.023	
Serious AE	22 (15.7)				16 (26.7)				0.070	

Abbreviations: AE, adverse event; ALT, alanine aminotransferase; AST, aspartate aminotransferase; SoraTACE, sorafenib plus transarterial chemoembolization.

Treatment delays due to AEs, including palmar-plantar erythrodysesthesia syndrome, thrombocytopenia, and liver function impairment, were less common in the SoraHAIC group (27 [19.3%] vs. 22 [36.7%]; *P =* 0.009). In total, 81 participants (40.5%) experienced AEs requiring dose reductions (56 [40.0%] vs. 25 [41.7%]; *P =* 0.83). The AEs that most frequently led to dose reduction were thrombocytopenia (*n* = 51), followed by palmar-plantar erythrodysesthesia syndrome (*n* = 44) and abdominal pain (*n* = 42). In the SoraHAIC group, two participants (1.4%) discontinued study treatment due to liver function impairment and subsequently received sorafenib monotherapy. In contrast, five participants (8.3%) in the SoraTACE group discontinued study treatment for the same reason and subsequently received sorafenib monotherapy (including two patients who subsequently and sequentially underwent HAIC treatment). The frequencies of dose reduction and discontinuation due to an AE or disease progression are shown in Supplementary Table 4.

### 3.4 Exploratory outcomes

**Fig. 3:**
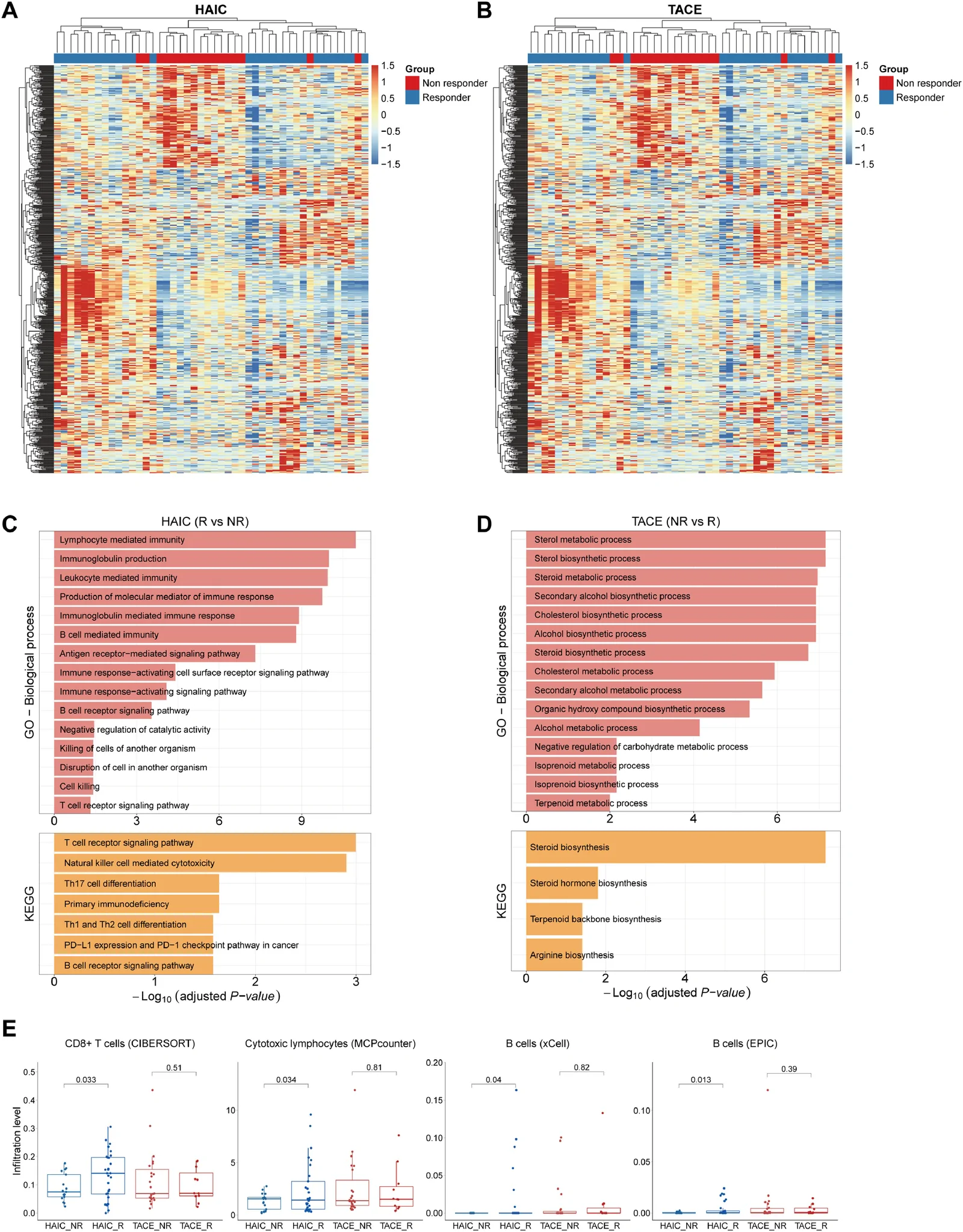
Transcriptomic characteristics of hepatocellular carcinoma tissues across different groups. (A, B) Heatmaps of differentially expressed genes stratified by response status to HAIC (A) and TACE (B). (C) Functional enrichment analysis of genes upregulated in HAIC responders. (D) Functional enrichment analysis of the upregulated genes in TACE nonresponders. (E) Differential immune cell infiltration in responders and nonresponders to HAIC and TACE, respectively, as estimated by the indicated methods. HAIC, hepatic arterial infusion chemotherapy; NR, non-responder; R, responder; TACE, transarterial chemoembolization.

The genomic profiles, mutation landscapes, and average methylation levels were comparable across the groups. We performed RNA-seq analysis (HAIC, *n* = 46; TACE, *n* = 34) and identified 685 and 600 differentially expressed genes (DEGs) from the HAIC and TACE treatment groups, respectively (Fig. 3A and B, Supplementary Fig. 5). Functional enrichment analysis suggested that immune-related pathways were enriched in HAIC responders, whereas metabolic-related pathways were predominant in TACE non-responders (Fig. 3C and D). Consistently, increased infiltration levels of immunocytes, such as CD8^+^ T cells and B cells, were observed in HAIC responders (Fig. 3E).

**Fig. 4:**
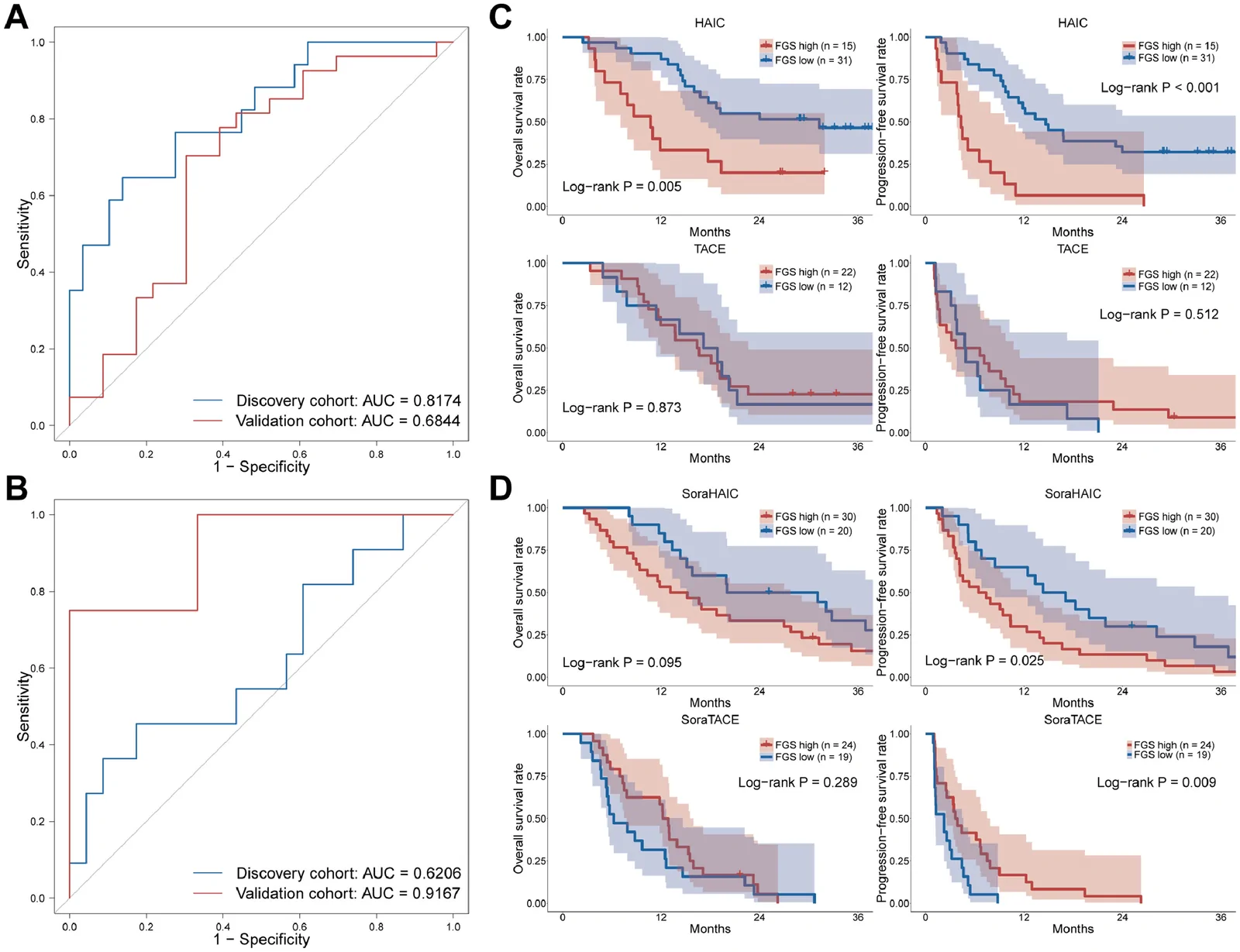
Predictive and prognostic value of the five-gene model. (A) ROC curves demonstrating the model's performance in predicting unfavorable outcomes after HAIC in both the discovery and validation cohorts. (B) ROC curves illustrating the model's performance in predicting favorable outcomes after TACE in both the discovery and validation cohorts. (C, D) Kaplan‒Meier curves for overall survival and progression-free survival in patients stratified by high and low five-gene scores in the discovery cohort (C) and validation cohort (D). The cutoff values were determined on the basis of the corresponding ROC analysis. HAIC, hepatic arterial infusion chemotherapy; ROC, receiver operating characteristic; Sora, sorafenib; TACE, transarterial chemoembolization.

Then we compared the signature scores of multiple gene sets extracted from patients with favorable or unfavorable outcomes in both treatment groups, identifying eight differentially enriched gene sets comprising 153 unique genes (Supplementary Table 5). Following this, a predictive model incorporating five genes—*PFKM, G6PD, ARSB, TSG101*, and *RAB11A*—was constructed using multivariate logistic regression in the discovery cohort (Supplementary Table 6). The model was applied to both the discovery and validation cohorts (SoraHAIC, *n* = 50; SoraTACE, *n* = 43), demonstrating that a higher score was associated with a worse prognosis in the HAIC group (area under curve [AUC] = 0.82 [95% CI: 0.69, 0.94]), whereas higher scores were associated with favorable outcomes in the TACE group (AUC = 0.62 [95% CI: 0.40, 0.84]) (Fig. 4).

**Fig. 5:**
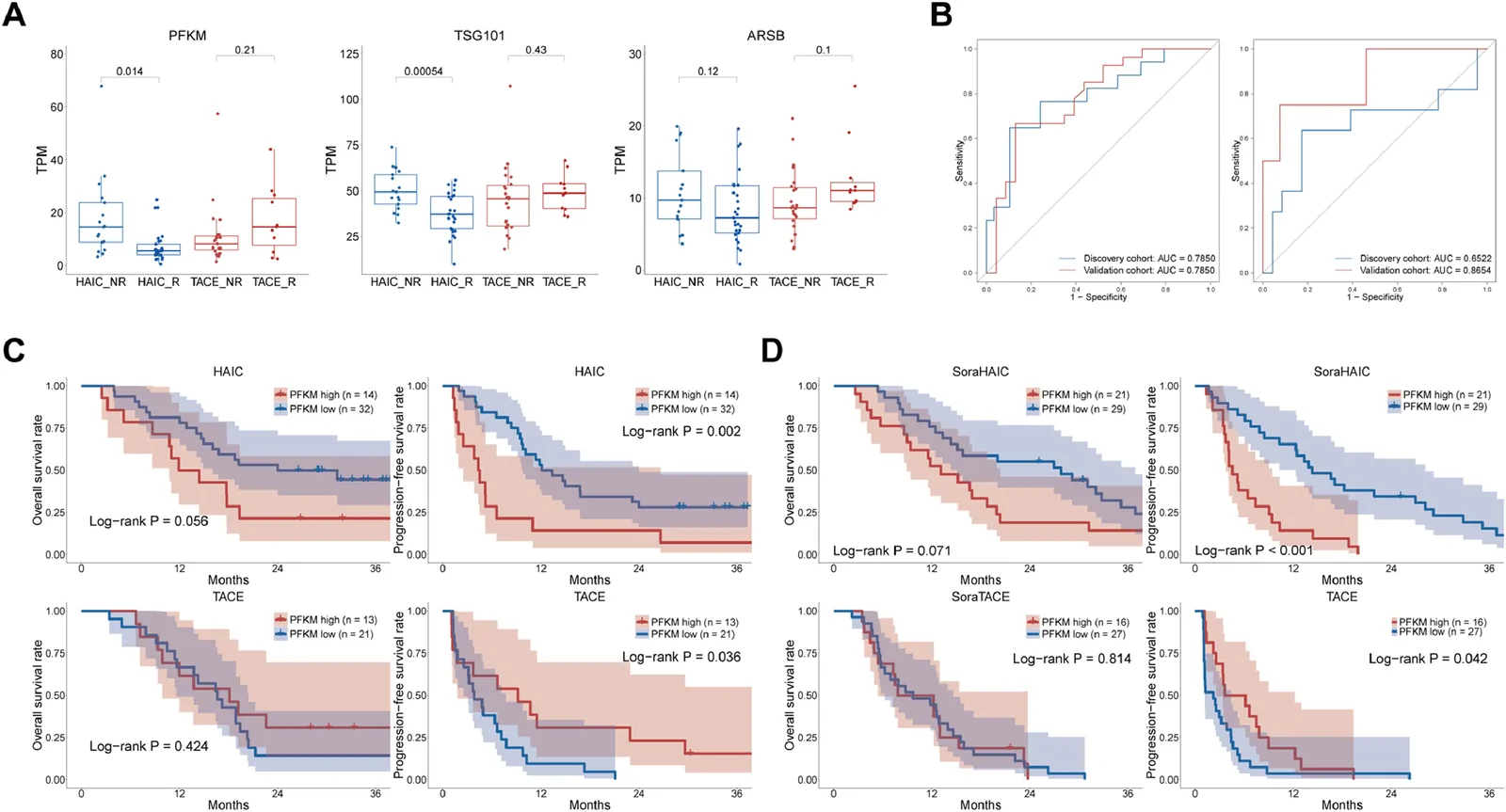
Predictive and prognostic value of PFKM. (A) Expression levels of PFKM, TSG101, and ARSB across different groups. (B) ROC curves demonstrating the model performance in predicting unfavorable outcomes after HAIC in both the discovery cohort (left panel) and the validation cohort (right panel). (C, D) Kaplan‒Meier curves for overall survival and progression-free survival in patients stratified by high and low PFKM expression levels in the discovery cohort (C) and validation cohort (D). The cutoff values were determined on the basis of the corresponding ROC analysis. HAIC, hepatic arterial infusion chemotherapy; ROC, receiver operating characteristic; Sora, sorafenib; TACE, transarterial chemoembolization.

We further investigated the methylation levels of the five genes in the predictive model. Among the 138 probes annotated to these genes (Supplementary Table 7), 11 probes corresponding to *PFKM, ARSB*, and *TSG101* exhibited differential methylation across the four groups (Supplementary Fig. 7). Elevated expression of *PFKM* and *TSG101* was associated with poorer outcomes in HAIC-treated patients, with *PFKM* expression positively correlated with the methylation level of cg10140784 (Fig. 5A and Supplementary Fig. 8). Moreover, analysis of the discovery cohort and the validation cohort indicated that higher *PFKM* levels were associated with better responses to TACE, whereas lower *PFKM* levels were predictive of favorable outcomes in HAIC-treated patients (Fig. 5B–D). These findings were further validated via qPCR analysis of tumor biopsies from the validation cohort (Supplementary Fig. 9). The detailed exploratory outcomes were provided in the Supplementary Materials.

## 4 Discussion

The primary results from our phase III trial revealed significantly improved OS and PFS with SoraHAIC over SoraTACE in advanced HCC participants. Furthermore, the clinical benefit was generally consistent across most subgroups, and SoraHAIC had a significantly lower frequency of grade 3–4 AEs than did SoraTACE. Additionally, participants with high *PFKM* expression benefited more from SoraTACE.

Although sorafenib was a first-line treatment for advanced HCC, the disease prognosis was still poor for patients in the Asia–Pacific region for an extended period historically. Nevertheless, the median OS of Chinese patients with advanced HCC treated with sorafenib monotherapy had improved to 10.7–11.4 months driven by advancements in disease management, the integration of immunotherapy, and the expanded availability of subsequent-line therapies.^29^^,^^30^ Considering the tumor burden in the Chinese population is high and locoregional therapies are recommended for reducing the tumor burden,^4^^,^^5^ TACE has been listed as a first-line treatment for selected patients with BCLC stage C disease in China and other Asia–Pacific regions.^7^^,^^17^^,^^18^^,^^20^ In several studies, such as the phase III STAH trial, the safety and efficacy of SoraTACE in advanced HCC were explored to evaluate potential synergy. In the STAH trial, the primary endpoint was not achieved. This may be due to several factors. First, 69.4% of patients from the trial received previous TACE treatment in the sorafenib plus TACE group, suggesting that these patients did not benefit from TACE alone. Second, only 28.3% of patients with Vp3–4 and 35.7% of patients with extrahepatic spread were included in this trial.^13^ Finally, OS may be affected by subsequent treatments. However, several retrospective studies have suggested that SoraTACE is effective in selected patients with advanced HCC, and the phase III STAH trial revealed that the time to progression, PFS, and the tumor response rate were significantly better with SoraTACE than with sorafenib monotherapy.^13^, ^14^, ^15^, ^16^^,^^31^ Therefore, we selected SoraTACE as a control treatment in our study.

In this phase III study, the 42.0% lower risk of death and the significantly longer OS with SoraHAIC than with SoraTACE are underpinned by a 3.1-month increase in median PFS, a corresponding 52.0% decrease in the risk of disease progression or death, and an objective response rate of 46.8%. One possible reason for the encouraging results with respect to the antitumor activity of SoraHAIC is that FOLFOX-HAIC can provide stable local high concentrations of the chemical agents in the tumor for more than 24 hours, whereas most chemotherapeutic agents delivered through TACE (even a specially formulated water-in-oil Lipiodol emulsion that is stable *in vitro*) can be released into the systemic circulation within less than one hour.^32^ Another possible reason is that repeated delivery of chemotherapeutic agents is allowed in Oxaliplatin + Leucovorin + 5-Fluorouracil (FOLFOX)-HAIC because HAIC (without embolization) can preserve the patency of the hepatic artery. In addition, a higher proportion of participants in the SoraHAIC group subsequently received PD-1 inhibitor therapy, which might contribute to improved outcomes. Our transcriptomic profiling analysis revealed that an immune-inflamed ("hot") tumor microenvironment (TME) phenotype—characterized by significantly elevated infiltration of CD8^+^ T cells and B cells—pre-existed in the SoraHAIC group prior to treatment initiation. This TME signature is notably associated with favorable clinical outcomes.^33^ Additionally, because the fluorouracil-mediated suppression of drug transporters and sorafenib can further inhibit the activity of multidrug resistance-associated transporters,^34^^,^^35^ the synergism between sorafenib, fluorouracil, and oxaliplatin in SoraHAIC might enhance its antitumor activity. Finally, owing to significant tumor shrinkage, the curative surgical resection rate was higher in the SoraHAIC group than in the SoraTACE group (10.6% vs. 3.0%), which contributed to the OS benefit.

Despite the consistent PFS, the median OS of participants treated with SoraHAIC in this study was better than that reported in a previous study (15.6 months vs. 13.4 months),^24^ which was probably because more participants in this study received subsequent treatment with anti-PD-1/PD-L1 antibodies, lenvatinib, and another second-line tyrosine kinase inhibitor (TKI) therapy. In addition, the percentage of participants with Vp3–4 in this study was lower than that in previous studies (50.4% vs. 80.8%). However, the median OS and PFS times of SoraTACE participants were worse than those of the phase III STAH patients (median OS, 11.2 months vs. 12.8 months; PFS, 4.2 vs. 5.2 months).^13^ This finding is more likely related to the higher frequency of tumors with larger diameters and the higher percentage of patients with macrovascular invasion who received SoraTACE in our study. Additionally, the OS of SoraTACE participants in our study was consistent with that reported in a retrospective study that included patients with PVTT (11.2 months vs. 11.0 months).^16^

Subanalyses of OS and PFS were performed, and SoraHAIC provided substantial clinical benefits in most subgroups. In particular, with respect to OS and PFS, participants without extrahepatic spread benefited more from SoraHAIC than do participants with extrahepatic spread, possibly because FOLFOX-HAIC is a locoregional therapy that is conducive to the control of intrahepatic lesions. In addition to extrahepatic spread (intersection *P =* 0.016), the neutrophil-to-lymphocyte ratio (NLR) may be predictive of improvements in the PFS of participants treated with SoraHAIC (intersection *P =* 0.013); however, the underlying mechanism remains unclear and needs further exploration.

The adverse events were consistent with those reported in previous studies.^24^ Participants treated with SoraTACE had significantly higher frequencies of adverse events related to embolization, including fever, elevated alanine aminotransferase, and elevated aspartate aminotransferase, whereas participants treated with SoraHAIC had a significantly higher frequency of hypoalbuminemia. Notably, the ORR per mRECIST in the SoraTACE group was 41% among participants who experienced ALT/AST elevation versus 28.6% in those who did not. In the SoraHAIC group, the corresponding ORRs were 67% and 59.2%, respectively. This pattern suggested that the association between transient hypertransaminasemia and objective tumor response might be more pronounced in the TACE group.^36^ The frequency of grade 3–4 AEs was significantly higher with SoraTACE than with SoraHAIC, which might explain why the number of treatment sessions in the SoraTACE group was lower and why more participants crossed over to SoraHAIC. These AEs are expected and manageable by treatment interruption or dose modification. Additionally, liver function deterioration after transarterial therapy, which can lead to the interruption or discontinuation of systemic treatment, is a significant adverse prognostic factor.^37^ Therefore, a thorough evaluation of tumor blood supply prior to HAIC or TACE and the use of superselective catheterization are crucial to reduce the risk of liver function deterioration.

Moreover, a comprehensive biomarker study was conducted to predict treatment response and determine the optimal treatment for patients with advanced HCC. Our results showed that patients who have a baseline active immune response in the tumor respond better to HAIC and that hypermetabolic tumors may respond better to TACE. These patients can be further identified as having high *PFKM* expression. *PFKM* is a member of the PFK family and assists in regulating glycolysis to promote tumor growth.^38^ *PFKM* has also been reported to promote resistance of tumors to bevacizumab.^39^ These findings indicate that TACE and antiangiogenic treatments, such as bevacizumab, may be an ideal combination.

This trial has several limitations. First, SoraTACE is not a first-line treatment for advanced HCC in Western countries, although this combined therapy has been listed as a major treatment option in most Asian countries. Currently, in addition to sorafenib, atezolizumab plus bevacizumab and lenvatinib are recommended as first-line treatments for unresectable HCC.^40^ Therefore, systemic treatments combined with locoregional therapy might need further optimization, and head-to-head comparisons of combined therapy versus atezolizumab plus bevacizumab or lenvatinib in patients with advanced HCC are needed in the future. Second, our study had an open-label design, and tumor response was assessed by investigators, which may introduce potential bias. The poststudy treatments might be influenced by investigator and participant decisions. The number of participants who received subsequent hepatic resection was higher with SoraHAIC than with SoraTACE. This could be explained by the higher ORR in the SoraHAIC group, resulting in more translation to resectable HCC. Third, although no significant differences in baseline characteristics were observed between the two groups, the use of tumor size alone as a stratification factor was suboptimal and linked with the lack of value of the subgroup analysis. Critical baseline imbalances in factors such as vascular invasion or extrahepatic spread could significantly influence prognostic outcomes. Fourth, we observed a significantly higher crossover rate in the SoraTACE group compared with the SoraHAIC group (19.7% vs. 0%, *P <* 0.001). This imbalance might stem from an elevated disease progression risk. Despite applying intention-to-treat analysis, crossover events could confound intergroup comparisons through mechanisms such as dilution of the SoraHAIC treatment effect, compromised baseline equipoise between groups, and biased survival analyses. Finally, the predominant etiology of HCC in our study was HBV. The efficacy and safety of SoraHAIC in HCC with different etiologies warrant further validation in confirmatory studies.

In summary, treatment with SoraHAIC is associated with significantly better OS and PFS than with SoraTACE in patients with advanced HCC. Patients with high expression of PFKM might benefit more from SoraTACE.

## CRediT author contribution statement

**Zhicheng Lai:** Data curation, Formal analysis, Software, Visualization, Validation, Investigation and Writing – original draft. **Zefeng Du:** Data curation, Formal analysis, Software, Visualization and Writing – original draft. **Lichang Huang:** Data curation, Formal analysis, Software and Visualization. **Qijiong Li:** Data curation and Formal analysis. **Yexing Huang, Li Xu** and **Wei Wei:** Investigation. **Yaojun Zhang** and **Minshan Chen:** Project administration. **Ming Shi:** Conceptualization, Supervision and Writing – review & editing. **Anna Kan:** Validation, Writing – original draft and Writing – review & editing. **Minke He:** Conceptualization, Validation, Supervision, Writing – review & editing.

## Ethics statement

This open-label, single-center, phase III clinical trial is registered at http://ClinicalTrials.gov (NCT02856126). Protocol approval was obtained from the institutional review board or ethics committee of Sun Yat-sen University of Cancer Center (approval number: B2016-025-01). This study was conducted in accordance with the International Conference on Harmonization guidelines for Good Clinical Practice and the principles of the Declaration of Helsinki. All patients provided written informed consent. The authors vouch for the accuracy and completeness of the data and for the fidelity of the trial to the protocol.

## Funding

This work was supported by 10.13039/501100012166National Key Research and Development Program of China (grant number: 2023YFA0915703), 10.13039/501100001809National Natural Science Foundation of China (grant numbers: 82272890, 82203126), Beijing iGandan Foundation (grant number: GDXZ-08-23), 10.13039/501100002858China Postdoctoral Science Foundation (grant number: 2023TQ0390), and Young Talents Program of Sun Yat-sen University Cancer Center (grant numbers: YTP-SYSUCC-0071, YTP-SYSUCC-0077).

## Declaration of competing interest

The authors declare that they have no known competing financial interests or personal relationships that could have appeared to influence the work reported in this paper.

## Appendix Supplementary materials

Image, application 1

Image, application 2

Image, application 3

Image, application 4
