# Supplementary material for: Sorafenib plus hepatic arterial infusion chemotherapy versus sorafenib plus transarterial chemoembolization in advanced hepatocellular carcinoma: a phase III trial and biomarker analyses
Source: J Natl Cancer Cent. 2026 May 19;6(4):410–9. doi: 10.1016/j.jncc.2026.04.002 (PMC13494611; doi:10.1016/j.jncc.2026.04.002)

Supplementary Materials

Sorafenib plus hepatic arterial infusion chemotherapy versus sorafenib plus transarterial chemoembolization in advanced HCC: a phase 3 trial and biomarker analyses

Zhicheng Lai, Zefeng Du, Lichang Huang, Qijiong Li, Yexing Huang, Li Xu, Wei Wei, Yaojun Zhang, Minshan Chen, Ming Shi, Anna Kan, Minke He

Supplementary method

Statistical analysis

Efficacy was assessed in all participants who had been randomly assigned to treatment (the intention-to-treat population). The results are reported as the means (standard deviations [SDs]), numbers (%), or medians (95% confidence intervals [CIs]) and were compared via Student's *t* tests or chi-square tests. Between-group comparisons of both overall survival (OS) and progression-free survival (PFS) were analyzed with a log-rank test, and hazard ratios for disease progression or death were estimated with a Cox proportional hazards model. Kaplan–Meier analysis was performed to evaluate OS and PFS. Confirmed response rates and differences were compared between treatment groups with the Cochran–Mantel–Haenszel test. All *P* values were two-sided, with *P* values less than 0.05 considered significant.

Whole-exome sequencing analysis

Fresh-frozen tumor tissues and matched peripheral blood samples were collected from each patient. Genomic DNA was extracted using the DNeasy Blood & Tissue Kit (Qiagen). DNA concentration was quantified using the Qubit 3.0 Fluorometer (Invitrogen), and samples meeting the criteria (DNA concentration ≥ 20 ng/μl and total DNA ≥ 0.4 μg) were selected for downstream processing. DNA (200 ng to 1 μg) was fragmented into 180-280 bp segments using the Covaris S2 ultrasonicator (Covaris).

Libraries were prepared using the Agilent SureSelect Human All Exon V5/V6 kit, following the manufacturer's instructions. The resulting libraries were quality-checked and sequenced on the Illumina HiSeq PE150 platform.

Raw sequencing reads underwent quality control to trim adapter sequences and remove poly(N) and low-quality reads using FASTP (v.0.23.2) (1) using default parameters. The processed FASTQ files were aligned to the human reference genome (hg38/GRCh38) using the Burrows-Wheeler Aligner (BWA, v0.7.17) (2) and sorted with SAMtools (v1.17) (3). Following alignment, the Genome Analysis Toolkit (GATK v4.4.0.0) best practice pipeline (4) was applied for PCR duplicate removal and base quality score recalibration. Somatic mutations, including single-nucleotide variants (SNVs) and small insertions/deletions (InDels), were identified using Mutect2. A Panel of Normal (PON) was first generated, and somatic mutations were called by comparing each sample with its matched peripheral blood sample and the PON file. Variants were filtered using GATK *FilterMutectCalls*, and only those flagged as 'PASS' were retained for further analysis. Finally, somatic mutations were annotated using GATK *Funcotator*.

Tumor ploidy and purity were estimated using FACETS (5) following the developers' guidance. Tumor mutational burden (TMB) was calculated by pyTMB (v1.3.0) (6) with default parameters. Mutational signatures, including single base substitution (SBS), dinucleotide substitution (DBS), and insertion/deletion (ID), were extracted using Sigminer (v2.3.0) (7). Both standard non-negative matrix factorization (NMF) and SigProfiler approaches were applied in parallel. Signatures supported by both methods (cosine similarity $\geq$ 0.90) were retained for downstream analysis. The extracted signatures were subsequently compared to the COSMIC v3.2 database, and the most similar COSMIC signatures were identified and used for visualization.

DNA methylation microarray experiment and data analysis

Genomic DNA was extracted from samples using the DNeasy Blood and Tissue Kit (Qiagen). DNA purity and concentration were quantified using the Qubit 3.0 Fluorometer. For each sample, 500 ng DNA was subjected to bisulfite converted using

the EZ DNA Methylation Kits (Zymo Research, USA). The converted DNA products were processed using the Infinium MethylationEPIC BeadChip (Illumina) following the manufacturer's protocol.

DNA methylation data were primarily analyzed using the Chip Analysis Methylation Pipeline (ChAMP) (8). Methylation levels were represented as β values. Probes were filtered based on the following criteria: detection P-value > 0.01, fewer than 3 beads in at least 10% of samples, non-CpG probes, multi-hit probes, and probes located on chromosomes X and Y. The resulting β value matrix was normalized using Beta-Mixture Quantile Normalization (BMIQ) to correct for type I and type II probe bias. Subsequently, batch effects caused by BeadChip slide and array were assessed using Singular Value Decomposition Analysis (SVA) (9) and corrected by Combat (9). CpG sites were annotated using the EPIC2.0 annotation file (v2.hg38). Differential methylated probes (DMPs) were identified using the *champ.DMP* function, with CpGs meeting the criteria of $|\Delta\beta| \geq 0.1$ and p-value ≤ 0.01 classified as DMPs.

RNA sequencing analysis

Raw FASTQ files were processed using FASTP (v.0.23.2) (1) to remove sequencing adapters and assess the sequencing quality. Filtered reads were aligned to the human reference genome (hg38) using STAR (10) with default parameters. Gene expression quantification was performed using featureCounts (11) to generate raw counts. For downstream analyses, raw counts were normalized and converted to transcripts per million (TPM) using a custom R script.

Genes expressed in more than 50% of samples within each cohort were used for differential expression analysis. Differentially expressed genes (DEGs) between groups were identified using the DESeq2 (12). The analysis was performed using raw count matrices derived from aligned sequencing reads as input.

Gene Ontology (GO) analysis and the Kyoto Encyclopedia of Genes and Genomes (KEGG) analyses of the DEGs were performed by R package clusterProfiler (13). This tool enabled functional enrichment analysis, identifying significantly enriched

biological processes, molecular functions, and pathways associated with the DEGs. Additionally, single-sample gene set enrichment analysis (ssGSEA) was performed to estimate the enrichment scores of specific gene sets within individual samples. To estimate tumor infiltrating immune cells using different methods, the R package IOBR (14) was utilized for analysis, which integrates multiple algorithms to comprehensively assess immune landscape and functional enrichment.

Signature scores of multiple gene sets were extracted from patients with favorable or unfavorable outcomes in both treatment groups, and eight differentially enriched gene sets comprising 153 unique genes were identified. After extracting these 153 candidate genes, we performed univariate and multivariate logistic regression analyses (hepatic arterial infusion chemotherapy [HAIC]-favorable vs. transarterial chemoembolization [TACE]-favorable) to identify significant predictors. The model was constructed based on the regression coefficients derived from the multivariate logistic regression model in the discovery cohort, generating a weighted linear predictor for each patient. For score stratification, receiver operating characteristic (ROC) curve analysis in the HAIC subgroup of the discovery cohort was used to determine the optimal cutoff based on the maximal Youden index. This optimal cutoff was then consistently applied to both the discovery cohort and the validation cohort.

Supplementary results

Efficacy

The subgroup analysis of OS is shown in Supplementary Fig. 1A. The results indicated that SoraHAIC was more beneficial in most subgroups. The subgroup analysis of PFS is shown in Supplementary Fig. 1B. The results indicated that participants with a neutrophil: lymphocyte ratio (NLR) greater than 3 (intersection $P = 0.004$) or without metastasis (intersection $P = 0.010$) derived greater benefit from SoraHAIC.

Safety

The SoraHAIC group had 24 patients (17.1%) and the SoraTACE group had 19 patients (31.7%) who withdrew due to intolerable adverse events (*P* = 0.022). Among SoraTACE patients discontinuing for intolerance, reasons included: liver function impairment (including massive ascites) in 5 patients; severe hand-foot syndrome in 4; renal impairment in 3 (with 1 case complicated by concurrent liver impairment and infection); severe gastrointestinal reactions in 2; tricytopenia in 2; upper gastrointestinal bleeding in 2; biliary injury in 1. Among SoraHAIC patients discontinuing for intolerance, reasons comprised: severe gastrointestinal reactions and hand-foot syndrome (5 patients each); tricytopenia (4 patients); liver impairment (2 patients); renal impairment (2 patients); upper gastrointestinal bleeding (2 patients); and single cases attributed to intolerable gingival bleeding, fever, abdominal pain, and peripheral neuropathy.

Exploratory outcomes

We initially examined the difference in somatic mutations between patients with unresectable HCC (uHCC) from our previous study who were responsive to treatment and those who were nonresponsive to treatment (HAIC, *n* = 35; TACE, *n* = 30). The genomic profiles and mutation landscapes were comparable across the groups (Supplementary Fig. 2). We subsequently performed a genome-wide methylation analysis on the tumor samples of these patients. The average methylation levels were not significantly different between responsive and nonresponsive uHCC patients (Supplementary Fig. 3). In total, 1,226 and 506 differentially methylated probes (DMPs) were identified in the nonresponsive samples of the HAIC and TACE groups, respectively. These included 1,077 and 357 hypermethylated CpGs, as well as 149 and 149 hypomethylated CpGs, in each treatment group (Supplementary Fig. 4).

Although the relationship between methylation status and gene expression is complex, identifying differential methylation profiles related to clinical phenotypes and uHCC prognosis is of significant interest. To this end, we performed RNA-seq analysis (HAIC, *n* = 46; TACE, *n* = 34) and identified 685 and 600 DEGs from the HAIC and TACE

treatment groups, respectively (Fig. 3A and B, Supplementary Fig. 5). Functional enrichment analysis suggested that immune-related pathways were enriched in HAIC responders, whereas metabolic-related pathways were predominant in TACE non-responders (Fig. 3C and D). Consistently, increased infiltration levels of immunocytes, such as $CD8^+$ T cells and B cells, were observed in HAIC responders (Fig. 3E).

To identify patients suitable for either HAIC or TACE, we aimed to develop a predictive model based on biomarkers. Considering the limited number of DEGs, we initially compared the signature scores of multiple gene sets extracted from patients with favorable or unfavorable outcomes in both treatment groups. Among the 223 genes, 8 were differentially enriched across the four groups, comprising 153 genes (Supplementary Fig. 6 and Supplementary Table 6). These genes were then subjected to univariate and multivariate logistic regression analyses, resulting in a predictive model incorporating five genes: *PFKM*, *G6PD*, *ARSB*, *TSG101*, and *RAB11A* (Supplementary Table 7). When applied to both the discovery and validation cohorts (SoraHAIC, n = 50; SoraTACE, n = 43), the model demonstrated that a higher score predicted a worse prognosis in the HAIC group (AUC, 0.82 [95% CI: 0.69, 0.94]), whereas higher scores were associated with favorable outcomes in the TACE group (AUC, 0.62 [95% CI: 0.4, 0.84]) (Fig. 3).

We further investigated the methylation levels of the five genes in the predictive model. Among the 138 probes annotated to these genes (Supplementary Table 5), 11 probes corresponding to *PFKM* (cg10140784 and cg10239804), *ARSB* (cg16560436, cg06066117, cg20336081, cg06383915, cg10858308, cg02582925, cg27361227, and cg07687324), and *TSG101* (cg12686661) exhibited differential methylation across the four groups (Supplementary Fig. 7). Elevated expression of *PFKM* and *TSG101* was associated with poorer outcomes in HAIC-treated patients, with *PFKM* expression positively correlated with the methylation level of cg10140784 (Fig. 5A and Supplementary Fig. 8). Moreover, analysis of the discovery cohort and the validation cohort indicated that higher *PFKM* expression was associated with better responses to TACE, whereas lower *PFKM* levels were predictive of favorable outcomes in HAIC-

treated patients (Fig. 5B-D). These findings were further validated via RT–PCR analysis of tumor biopsies from the validation cohort (Supplementary Fig. 9).

References

1. Chen S, Zhou Y, Chen Y, Gu J. fastp: an ultra-fast all-in-one FASTQ preprocessor. Bioinformatics. 2018;34(17):i884-i90. doi: 10.1093/bioinformatics/bty560.

2. Li H. Aligning sequence reads, clone sequences and assembly contigs with BWA-MEM2013 March 01, 2013:[arXiv:1303.3997 p.]. Available from: https://ui.adsabs.harvard.edu/abs/2013arXiv1303.3997L.

3. Danecek P, Bonfield JK, Liddle J, et al. Twelve years of SAMtools and BCFtools. Gigascience. 2021;10(2):giab008. doi: 10.1093/gigascience/giab008.

4. McKenna A, Hanna M, Banks E, et al. The Genome Analysis Toolkit: a MapReduce framework for analyzing next-generation DNA sequencing data. Genome Research. 2010;20(9):1297-303. doi: 10.1101/gr.107524.110.

5. Shen R, Seshan VE. FACETS: allele-specific copy number and clonal heterogeneity analysis tool for high-throughput DNA sequencing. Nucleic Acids Res. 2016;44(16):e131. doi: 10.1093/nar/gkw520.

6. Dupain C, Gutman T, Girard E, et al. Tumor mutational burden assessment and standardized bioinformatics approach using custom NGS panels in clinical routine. BMC Biology. 2024;22(1):43. doi: 10.1186/s12915-024-01839-8.

7. Wang S, Li H, Song M, et al. Copy number signature analysis tool and its application in prostate cancer reveals distinct mutational processes and clinical outcomes. PLoS Genet. 2021;17(5):e1009557. doi: 10.1371/journal.pgen.1009557.

8. Morris TJ, Butcher LM, Feber A, et al. ChAMP: 450k Chip Analysis Methylation Pipeline. Bioinformatics (Oxford, England). 2014;30(3):428-30. doi: 10.1093/bioinformatics/btt684.

9. Johnson WE, Li C, Rabinovic A. Adjusting batch effects in microarray expression data using empirical Bayes methods. Biostatistics. 2007;8(1):118-27.

10. Dobin A, Davis CA, Schlesinger F, et al. STAR: ultrafast universal RNA-seq aligner. Bioinformatics. 2013;29(1):15-21. doi: 10.1093/bioinformatics/bts635.

11. Liao Y, Smyth GK, Shi W. featureCounts: an efficient general purpose program for assigning sequence reads to genomic features. Bioinformatics. 2014;30(7):923-30. doi: 10.1093/bioinformatics/btt656.

12. Love MI, Huber W, Anders S. Moderated estimation of fold change and dispersion for RNA-seq data with DESeq2. Genome Biology. 2014;15(12):550.

13. Xu S, Hu E, Cai Y, et al. Using clusterProfiler to characterize multiomics data. Nature Protocols. 2024. doi: 10.1038/s41596-024-01020-z.

14. Zeng D, Ye Z, Shen R, et al. IOBR: Multi-Omics Immuno-Oncology Biological Research to Decode Tumor Microenvironment and Signatures. Frontiers In Immunology. 2021;12:687975. doi: 10.3389/fimmu.2021.687975.

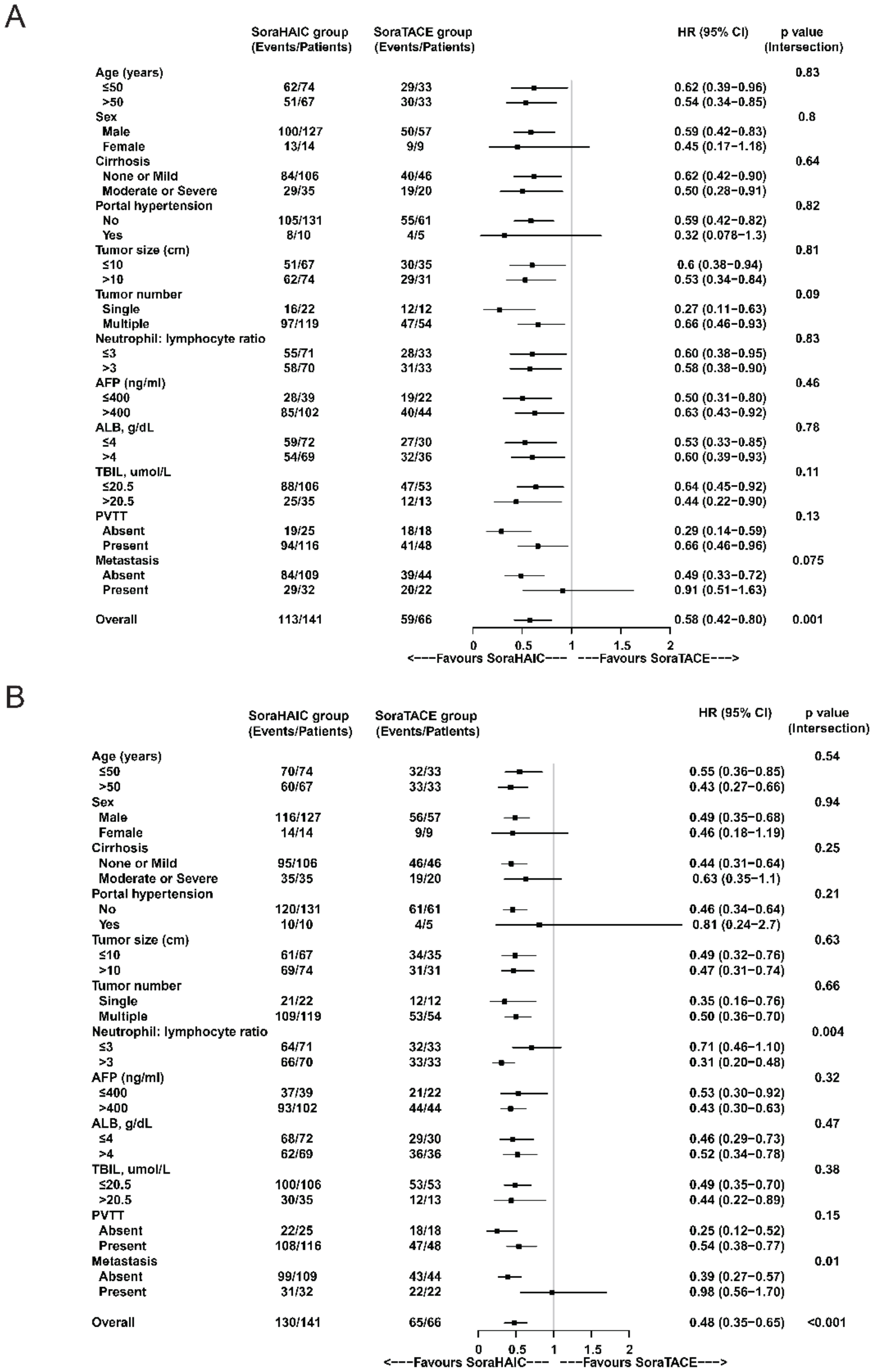

Supplementary Fig. 1. Subgroup analysis. (A) Forest plot of overall survival and progression-free survival (B). Each point represents the HR of each subgroup, and the horizontal line represents the 95% CI of the HR. The vertical line indicates an HR=1. AFP, alpha-fetoprotein; ALB, albumin; CI, confidence interval; HAIC, hepatic arterial infusion chemotherapy; HR, hazard ratio; PVTT, portal vein tumor thrombus; Sora, sorafenib; TACE, transarterial chemoembolization; TBIL, total bilirubin.

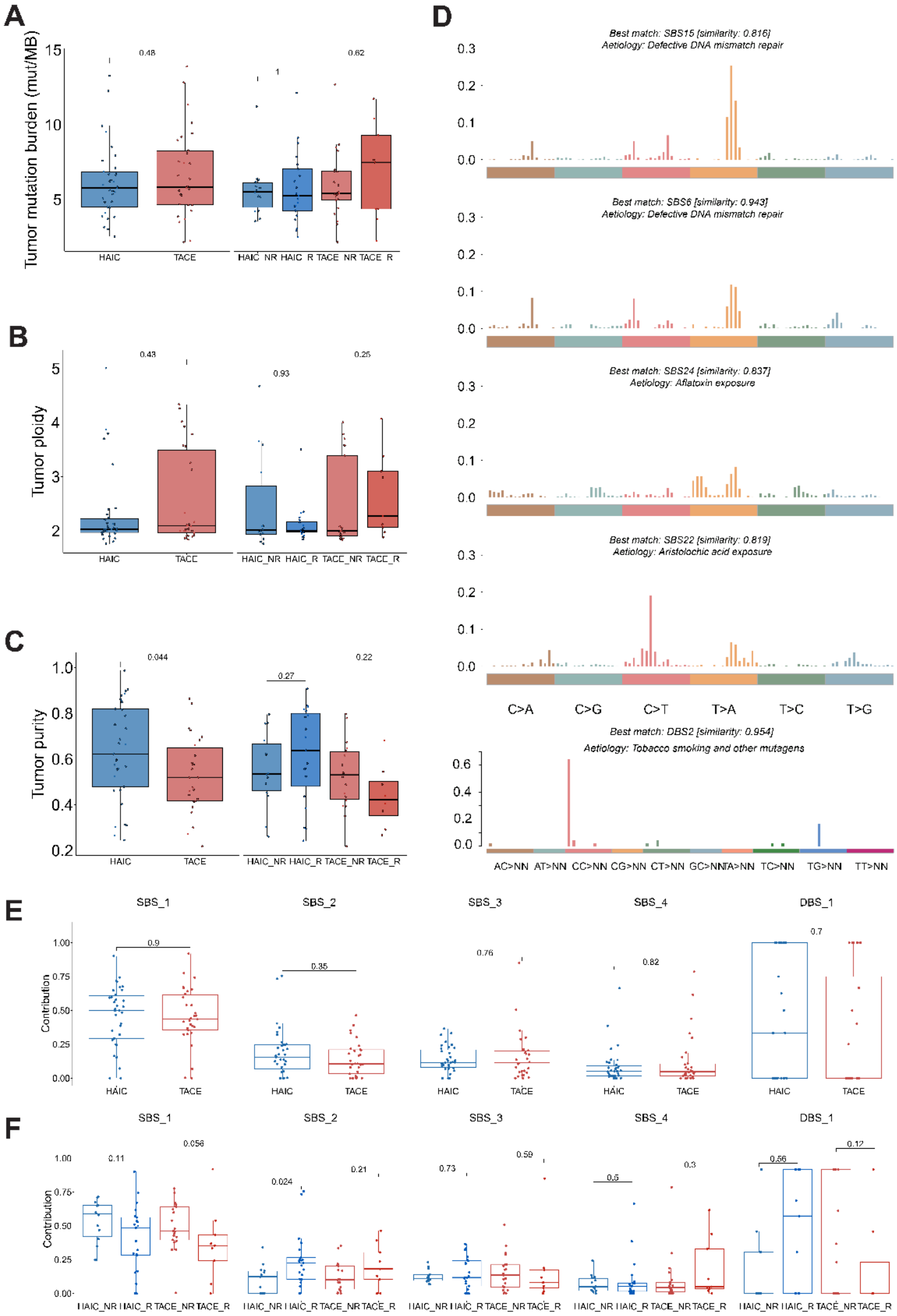

Supplementary Fig. 2. Genomic characteristics of HCC tissues across different groups. (A-C) The tumor mutation burden (A), tumor ploidy (B), and tumor purity (C) in the treatment groups stratified by response status. (D) Etiologies inferred from mutational signatures. (E-F) Relative contributions of the indicated mutational signatures in each group. HAIC, hepatic arterial infusion chemotherapy; HCC, hepatocellular carcinoma; NR, not response; TACE, transarterial chemoembolization; R, response.

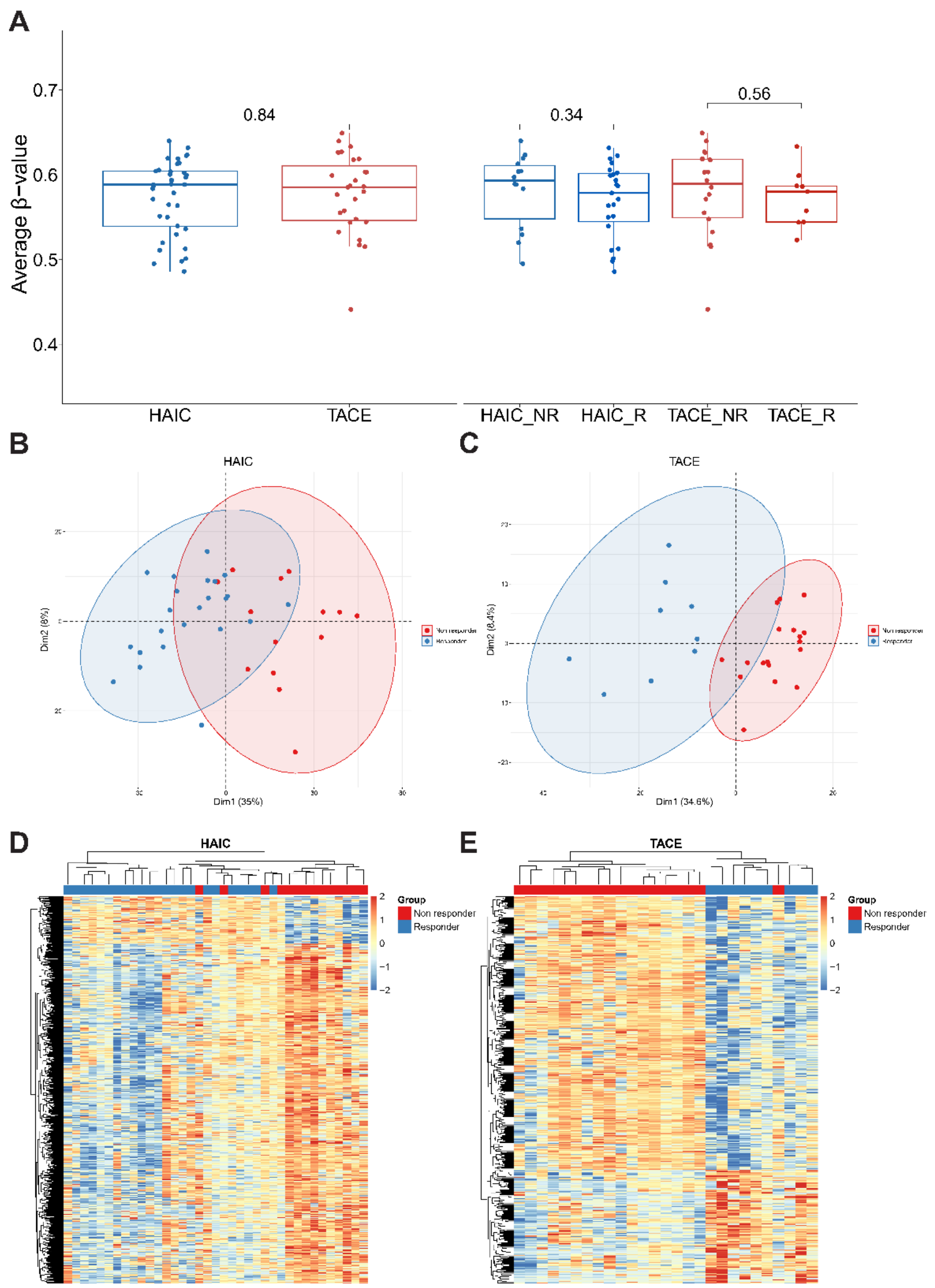

Supplementary Fig. 3. Methylation characteristics of genes in HCC tissues across different groups. (A) Average methylation levels stratified by treatment and response status. (B, C) Principal component analysis of differentially methylated probes based on response status in the HAIC (B) and TACE (C) groups. (D, E) Heatmaps displaying DMPs stratified by response status in the HAIC (D) and TACE (E) groups. HAIC, hepatic arterial infusion chemotherapy; HCC, hepatocellular carcinoma; NR, not response; TACE, transarterial chemoembolization; R, response.

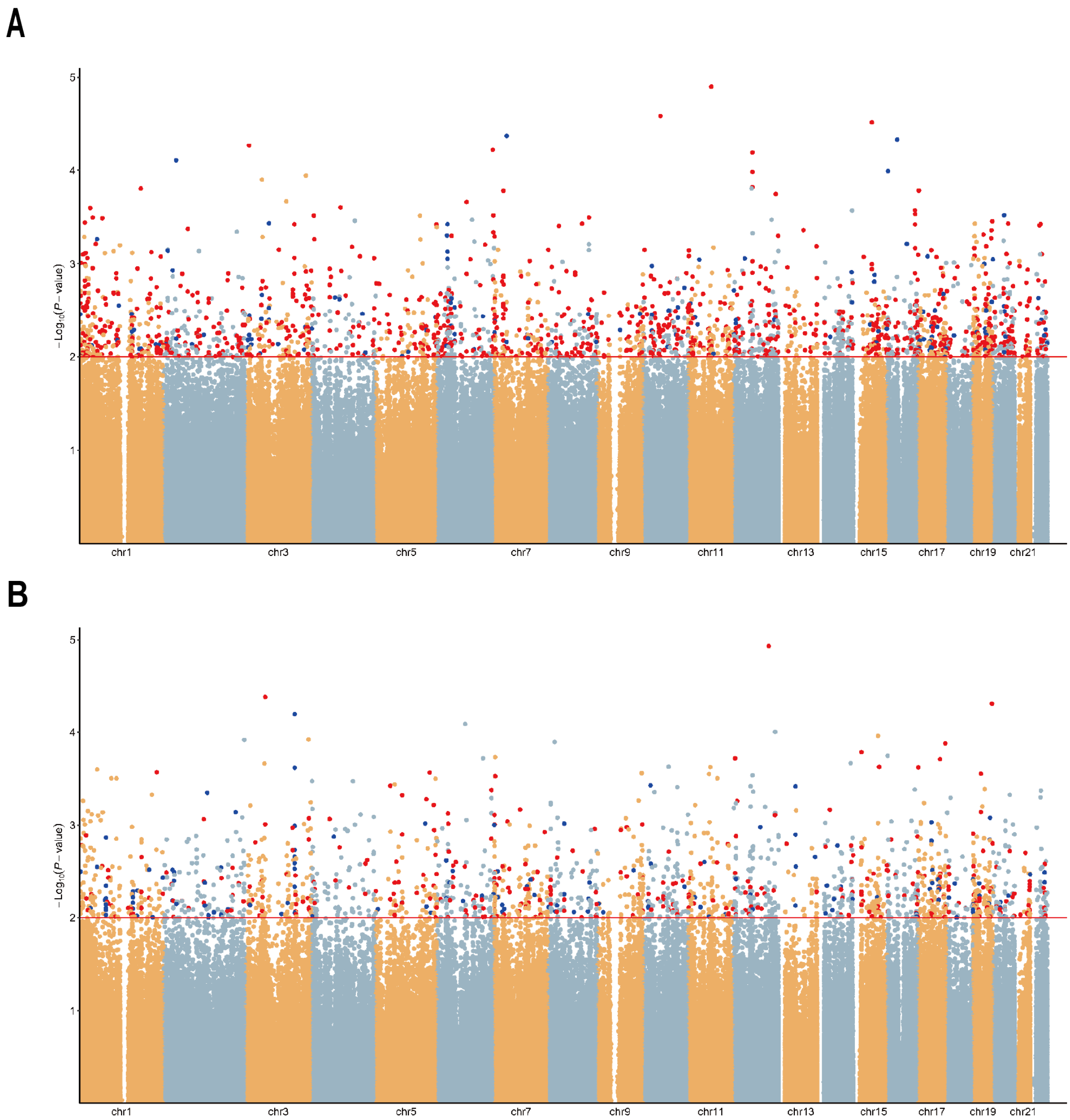

Supplementary Fig. 4. Manhattan plot of genome-wide DNA methylation analysis in responders and nonresponders to HAIC and TACE. (A, B) The 1,226 differentially methylated probes are highlighted in blue (hypomethylated) or red (hypermethylated) in nonresponders of the HAIC (A) and TACE (B) groups. Each point represents a CpG site, which is plotted according to its chromosomal location. HAIC, hepatic arterial infusion chemotherapy; TACE, transarterial chemoembolization.

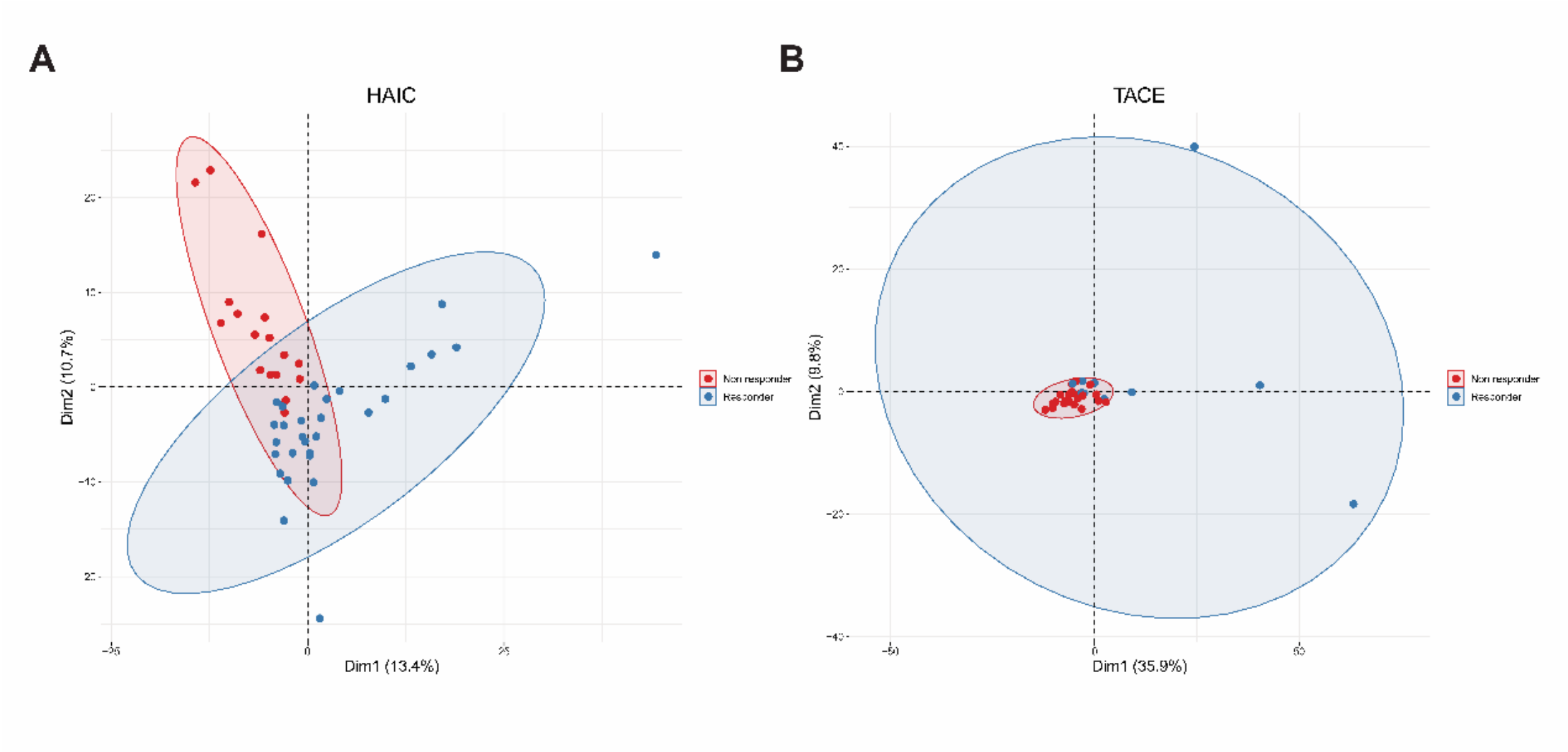

Supplementary Fig. 5. (A, B) Principal component analysis of differentially expressed genes stratified by response status to HAIC (A) and TACE (B). HAIC, hepatic arterial infusion chemotherapy; TACE, transarterial chemoembolization.

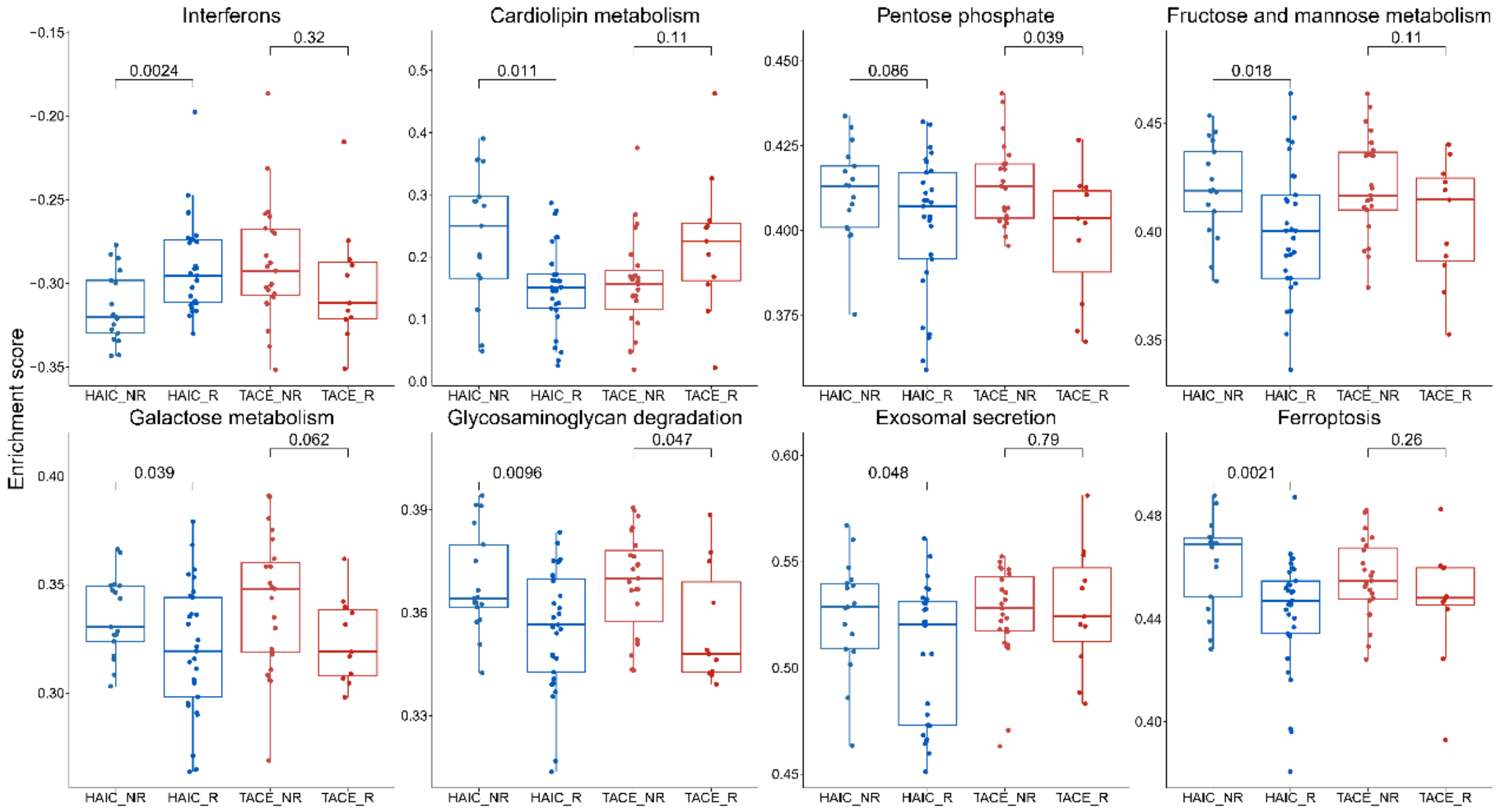

Supplementary Fig. 6. Differential enrichment of gene sets across the four indicated groups.

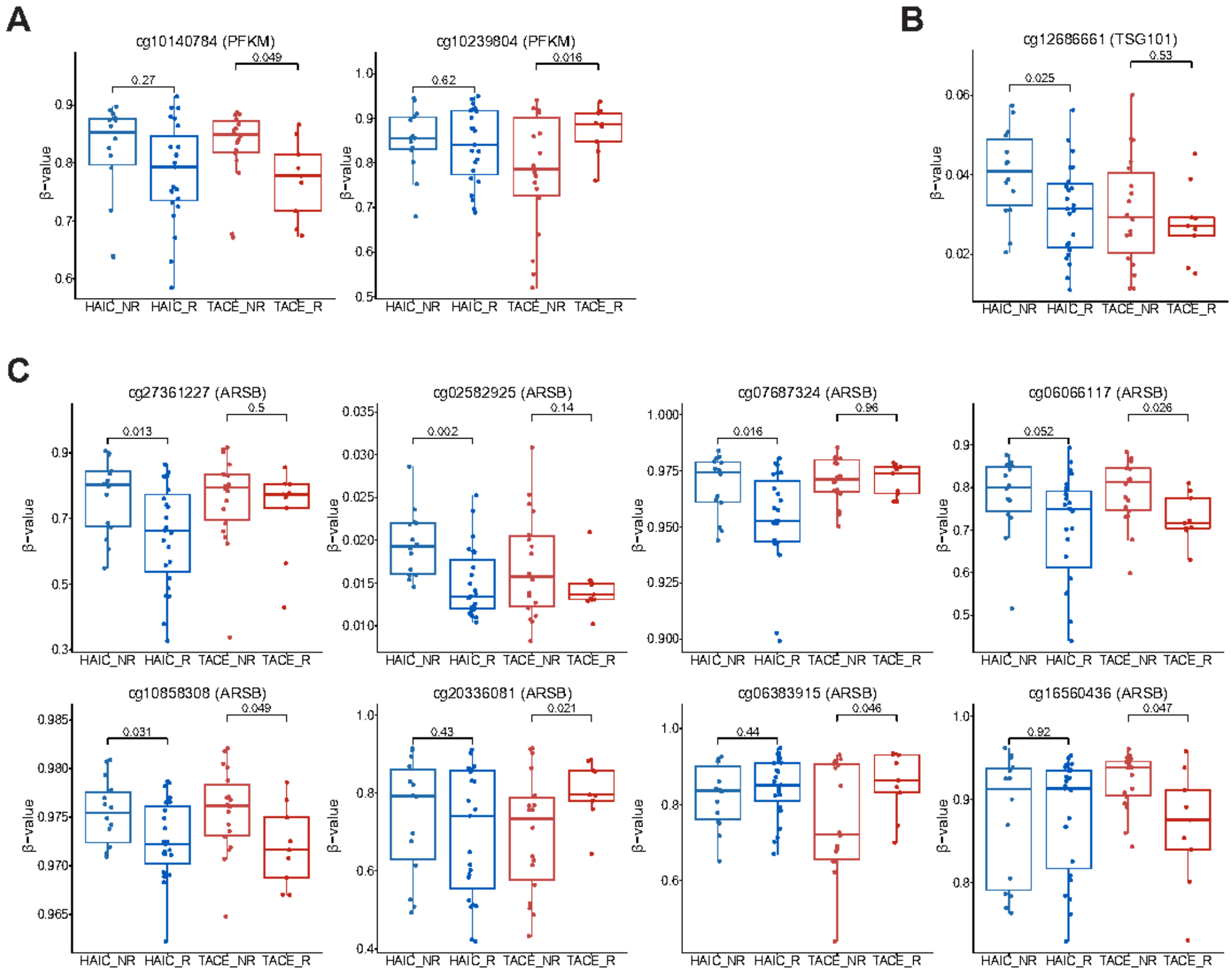

Supplementary Fig. 7. (A-C) Differential methylation of probes annotated to PFKM (A), TSG101 (B), and ARSB (C), stratified by response status.

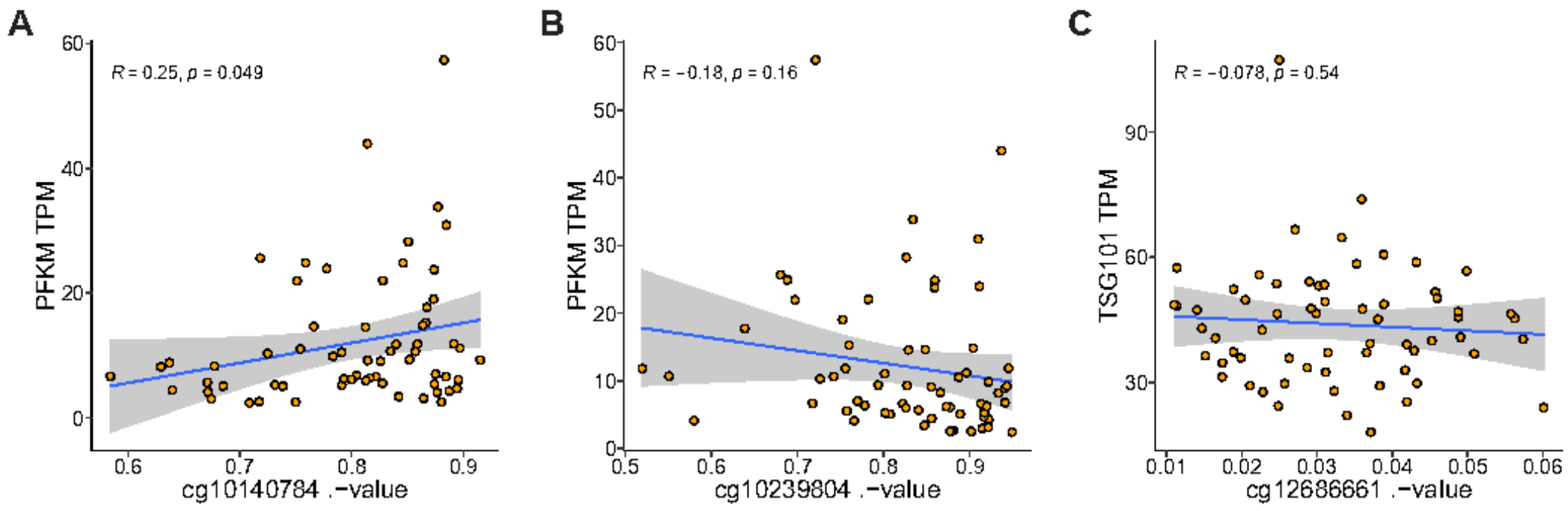

Supplementary Fig. 8. Correlation analysis between gene expression and methylation levels. (A) Correlation between PFKM expression and the cg10140784 methylation level. (B) Correlation between PFKM expression and the cg10239804 methylation level. (C) Correlation between TSG101 expression and the cg12686661 methylation level.

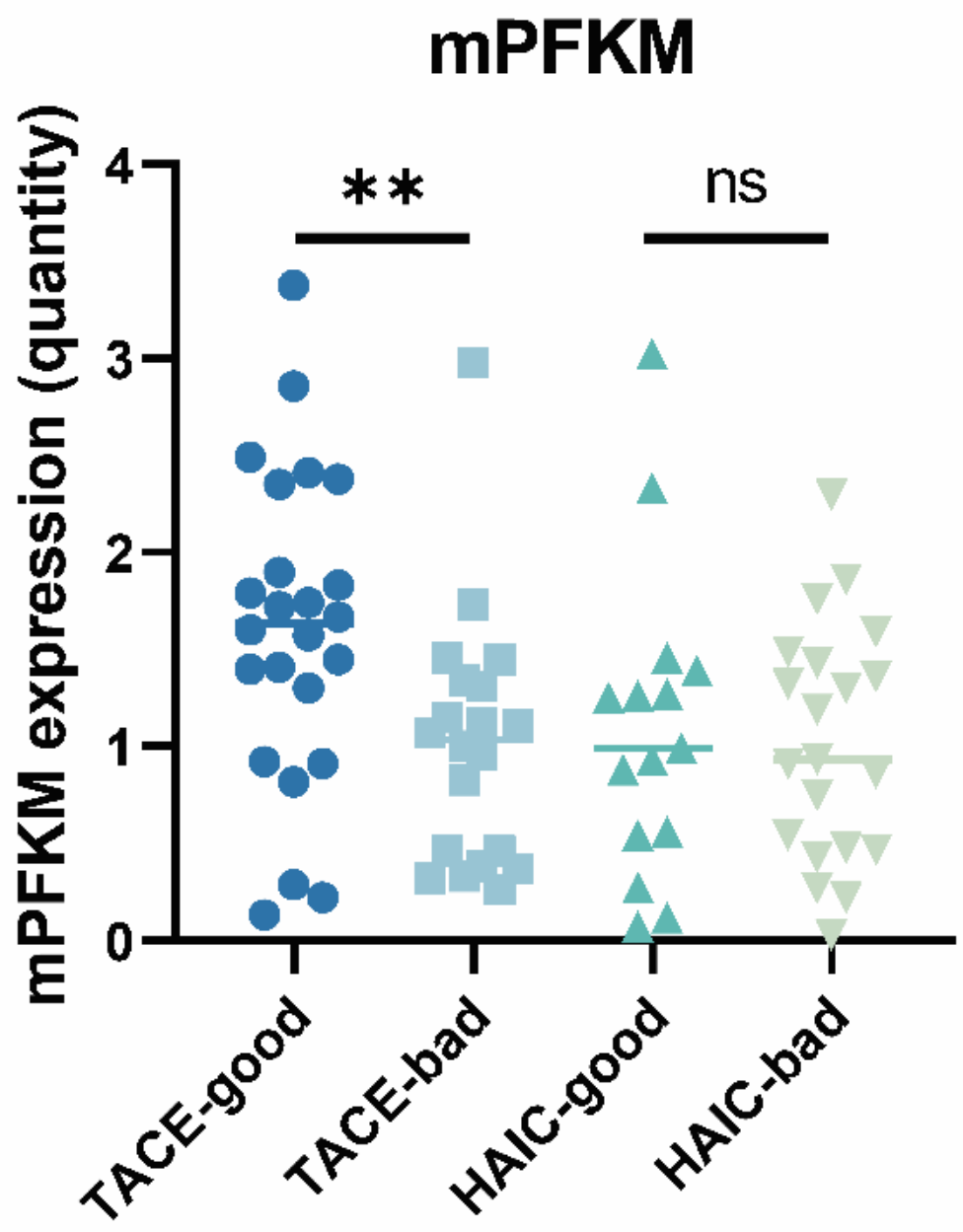

Supplementary Fig. 9. Detection of PFKM mRNA expression levels by RT-PCR in different groups of the validation cohort. HAIC, hepatic arterial infusion chemotherapy; TACE, transarterial chemoembolization.

Supplementary Table 1

Treatment administration.

	SoraHAIC group (*n* = 141)	SoraTACE group (*n* = 66)	*P*
Study treatment			
HAIC/TACE cycle, median (range)	4 (1-8)	1 (1-5)	< 0.001
Duration of sofafenib, months	4.8	3.4	< 0.001
Crossover to SoraHAIC/SoraTACE, No.	0	13	< 0.001
Number of patients who received subsequent treatments, No. (%)			
Sorafenib	26 (18.4)	20 (30.3)	0.056
HAIC	20 (14.2)	17 (25.8)	0.043

TACE	11 (7.8)	13 (19.7)	0.013
Liver transplantation	2 (1.4)	0	0.210
Resection	15 (10.6)	2 (3.0)	0.063
Ablation	13 (9.2)	3 (4.5)	0.240
Radiotherapy	19 (13.5)	5 (7.6)	0.220
TKI	32 (22.7)	10 (15.2)	0.210
Chemotherapy	2 (1.4)	0	0.210
PD-1 antibody	20 (14.2)	5 (7.6)	0.170

Abbreviations: HAIC, hepatic arterial infusion chemotherapy; Sora, sorafenib; TACE, transarterial chemoembolization; TKI, tyrosine kinase inhibitor.

Supplementary Table 2

Univariate and multivariate analysis of OS and PFS.

Subsets	Overall survival			Progression-free survival		
	Univariate analysis	Multivariate analysis		Univariate analysis	Multivariate analysis	
	P_1	HR (95% CI)	P_2	P_1	HR (95% CI)	P_2
Age (> 50 vs ≤ 50, years)	0.088	0.75 (0.55-1.00)	0.071	0.350		
Sex (male vs female)	0.081	0.80 (0.50-1.30)	0.340	0.970		
ECOG (1-2 vs 0)	0.002	2.00 (1.40-2.90)	2.5^10^{-4}	0.650		
Cirrhosis (moderate or severe vs none or mild)	0.061	1.60 (1.10-2.20)	0.012	0.100		

Portal hypertension (yes vs no)	0.570			0.580		
Tumor size (> 10 vs ≤ 10, cm)	0.590			0.650		
Tumor number (multiple vs single)	0.220			0.074	1.40 (0.95-2.00)	0.094
NLR (> 3 vs ≤ 3)	0.720			0.450		
HBsAg (positive vs negative)	0.440			0.310		
AFP (> 400 vs ≤ 400, ng/ml)	0.015	1.40 (1.00-2.00)	0.042	0.190		
ALB (< 4 vs ≥ 4, g/dL)	0.130			0.800		
TBIL (> 20.5 vs ≤ 20.5, μmol/L)	0.880			0.430		
ALBI grade (2 vs 1)	0.230			0.690		
PVTT (present vs absent)	0.670			0.720		

Metastasis (present vs absent)	0.057	1.10 (0.78-1.60)	0.530	4.6^10^{-6}	1.50 (1.00-2.00)	0.027
Group (SoraHAIC vs SoraTACE)	7.9^10^{-4}	0.49 (0.35-0.70)	6.0^10^{-5}	3.1^10^{-6}	0.52 (0.38-0.71)	5.5^10^{-5}

Abbreviations: ALBI, albumin-bilirubin grade; ALB, albumin; AFP, alpha-fetoprotein; CI, confidence interval; ECOG, Eastern Cooperative Oncology Group; HBsAg, hepatitis B surface antigen; HAIC, hepatic arterial infusion chemotherapy; HR, hazard ratio; NLR, neutrophil-to-lymphocyte ratio; PVTT, portal vein tumor thrombus; Sora, sorafenib; TACE, transarterial chemoembolization; TBIL, total bilirubin.

Supplementary Table 3

Baseline characteristics of patients achieving complete response according to mRECIST.

Patients ID	Group	Age	Sex	Tumor diameter, cm	Tumor numbers	PVTT	HVTT	Extrahepatic spread
1	SoraHAIC	43	Male	10.9	Multiple	Vp1-2	Absent	Present
2	SoraHAIC	40	Male	3.1	Single	Vp1-2	Absent	Absent
3	SoraHAIC	56	Male	6.4	Single	Vp3	Absent	Absent
4	SoraHAIC	46	Male	9.9	Multiple	Vp3	Absent	Absent
5	SoraHAIC	41	Male	10.8	Multiple	Vp1-2	Absent	Absent
6	SoraTACE	56	Male	11.5	Multiple	Vp1-2	Absent	Present
7	SoraHAIC	70	Male	5.6	Multiple	Vp1-2	Present	Absent

8	SoraHAIC	39	Male	13.9	Multiple	Absent	Present	Absent
9	SoraHAIC	70	Male	10.0	Multiple	Absent	Present	Absent
10	SoraHAIC	61	Female	6.4	Single	Vp1-2	Absent	Absent
11	SoraHAIC	49	Male	10.4	Multiple	Vp3	Present	Absent
12	SoraTACE	70	Male	9.6	Multiple	Vp1-2	Absent	Present
13	SoraTACE	24	Male	9.9	Multiple	Vp1-2	Absent	Present

Abbreviations: HAIC, hepatic arterial infusion chemotherapy; HVTT, hepatic vein tumor thrombus; mRECIST, modified Response Evaluation Criteria in Solid Tumors; PVTT, portal vein tumor thrombus; Sora, sorafenib; TACE, transarterial chemoembolization.

Supplementary Table 4

Study treatment dose reduction, delay, and discontinuation due to the disease progression or adverse events.

Subsets, No. (%)	SoraHAIC group (*n* = 140)	SoraTACE (*n* = 60)	*P*
Dose reduction	56 (40.0)	25 (41.7)	0.830
Sorafenib decrease	34 (24.3)	17 (28.3)	
Oxaliplatin dose decreased to 65 mg/m^2	24 (17.1)	-	
5FU dose decreased to 200 mg/m^2 bolus and 1200 mg/m^2	23 (16.4)	-	
Lipiodol decrease	-	15 (25.0)	
Epirubicin or lobaplatin decrease	-	2 (3.3)	
Delay	27 (19.3)	22 (36.7)	0.009
Within 30 days	23 (16.4)	17 (28.3)	
More than 30 days	4 (2.9)	5 (8.3)	
Discontinuation			
Disease progression	80 (57.1)	27 (45.0)	0.120
Intolerable AE	24 (17.1)	19 (31.7)	0.022

Abbreviations: HAIC, hepatic arterial infusion chemotherapy; TACE, transarterial chemoembolization. Statistical significance was assessed with the chi-square test.

Supplementary Table 8

Representativeness of study participants.

Cancer type	Hepatocellular carcinoma (HCC)
Considerations related to:	
Sex	In 2020, liver cancer was more prevalent in males than in females globally. According to GLOBOCAN, an estimated 632,320 males were diagnosed, with an age-standardized incidence rate of 14.1 per 100,000, compared to 273,357 females at a rate of 5.2 per 100,000.<br>This gender disparity is even more pronounced in China, where males accounted for approximately 74% of liver cancer cases. Data from the National Cancer Registry in China shows that the age-standardized incidence rate for males (26.7 per 100,000) was more than three times higher than that for females (8.7 per 100,000).
Age	According to database of the National Cancer Registry in China, liver cancer is most frequently diagnosed in males between 60 and 79 years old, accounting for nearly 45% of cases. This is followed by men aged 45 to 59 (37.3%) and 15 to 44 (10.1%). In contrast, liver cancer is less common in females under 60. Most female diagnoses occur at age 60 and above, with 53,400 cases in women aged 60 to 79 and 17,800 in those over 80.
Race/ethnicity	According to the GLOBOCAN 2020 database, an estimated 905,677 people were diagnosed with liver cancer worldwide

	in 2020, with an age-standardized incidence rate of 95 per 100,000. Incidence rates varied significantly by region. The highest rates were found in Eastern Asia (17.8 per 100,000) and Northern Africa (15.2 per 100,000). Conversely, the lowest rates occurred in South-Central Asia (3.0 per 100,000) and both Central and Eastern Europe and South America (4.3 per 100,000 for both regions). China contributed to a substantial portion of these cases, accounting for nearly half of all global diagnoses. In 2016, for example, the country had an estimated 388,800 new cases, with an age-standardized rate of 17.8 per 100,000.
Geography	In China, the incidence of liver cancer shows a clear geographic disparity. Rural areas have a higher age-standardized rate (19.3 per 100,000) than urban areas (16.3 per 100,000). This rural-urban difference is also seen when looking at specific regions. Southern rural areas have the highest incidence rate at 30.0 per 100,000, while Northern urban areas have the lowest rate at 10.5 per 100,000.
Other considerations	Over two-thirds of global liver cancer cases linked to HBV infection are found in less developed countries, with a significant concentration in China.
Overall representativeness of this study	The demographic characteristics of our study, including a lower median age of 50 and a higher male-to-female ratio, align with established trends for HCC. The gender distribution is consistent with findings from both the GLOBOCAN 2020 and the China National Cancer Registry. As our patients were mainly from major medical centers in

	southern China, the study cohort is largely representative of the national HCC population.

Representative images

SoraHAIC Patient 01

Before treatment

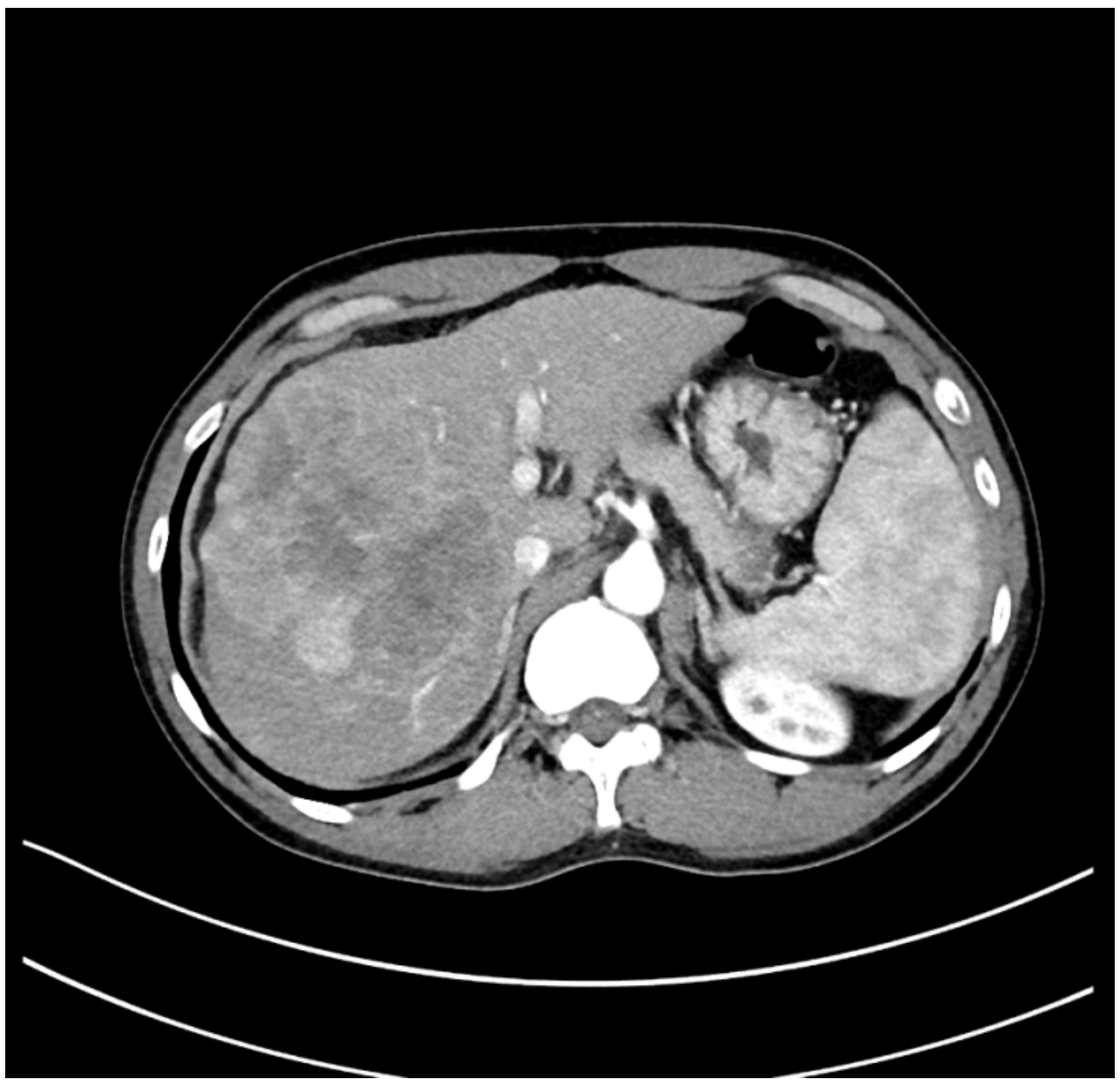

After treatment

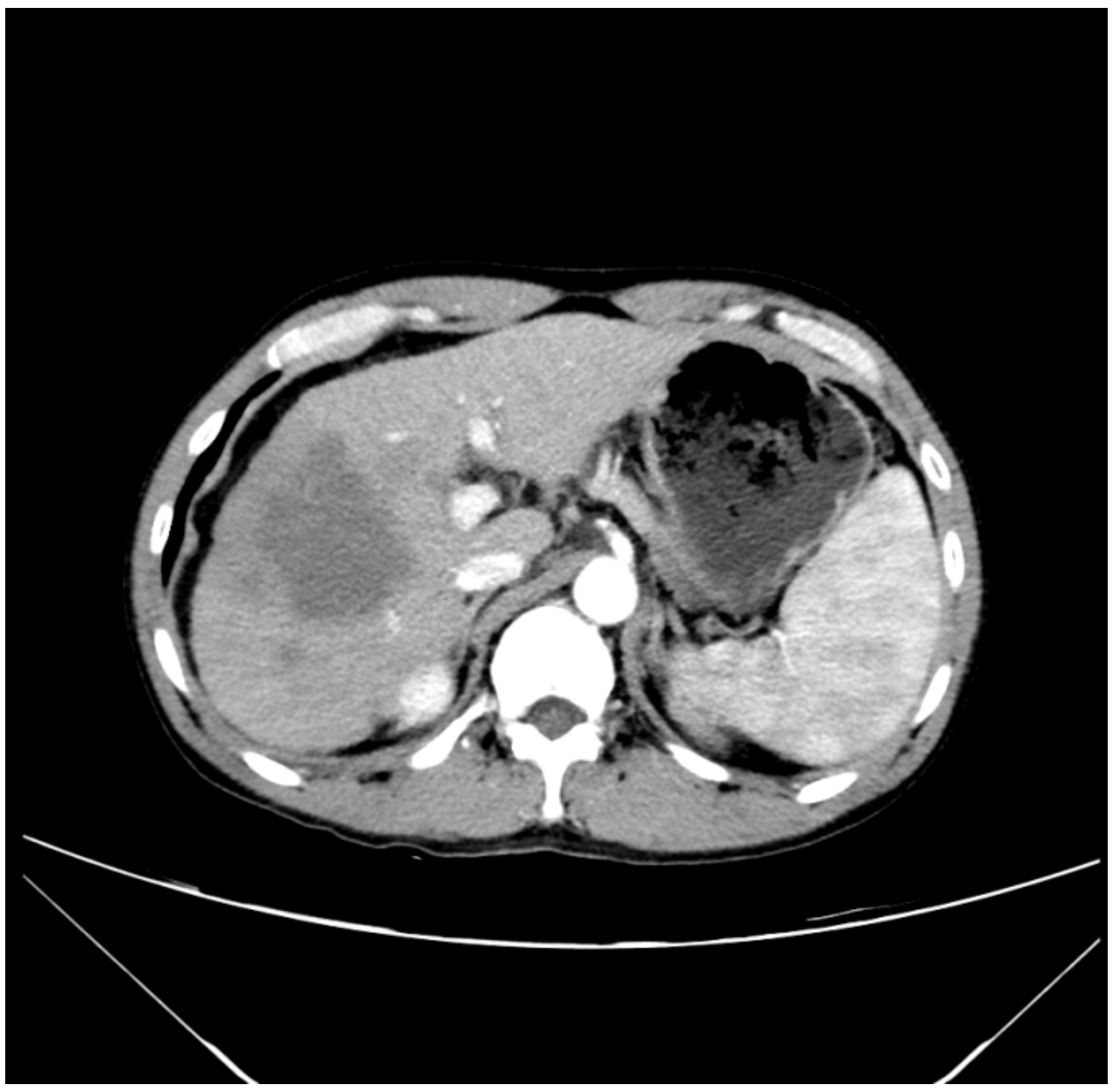

SoraHAIC Patient 02

Before treatment

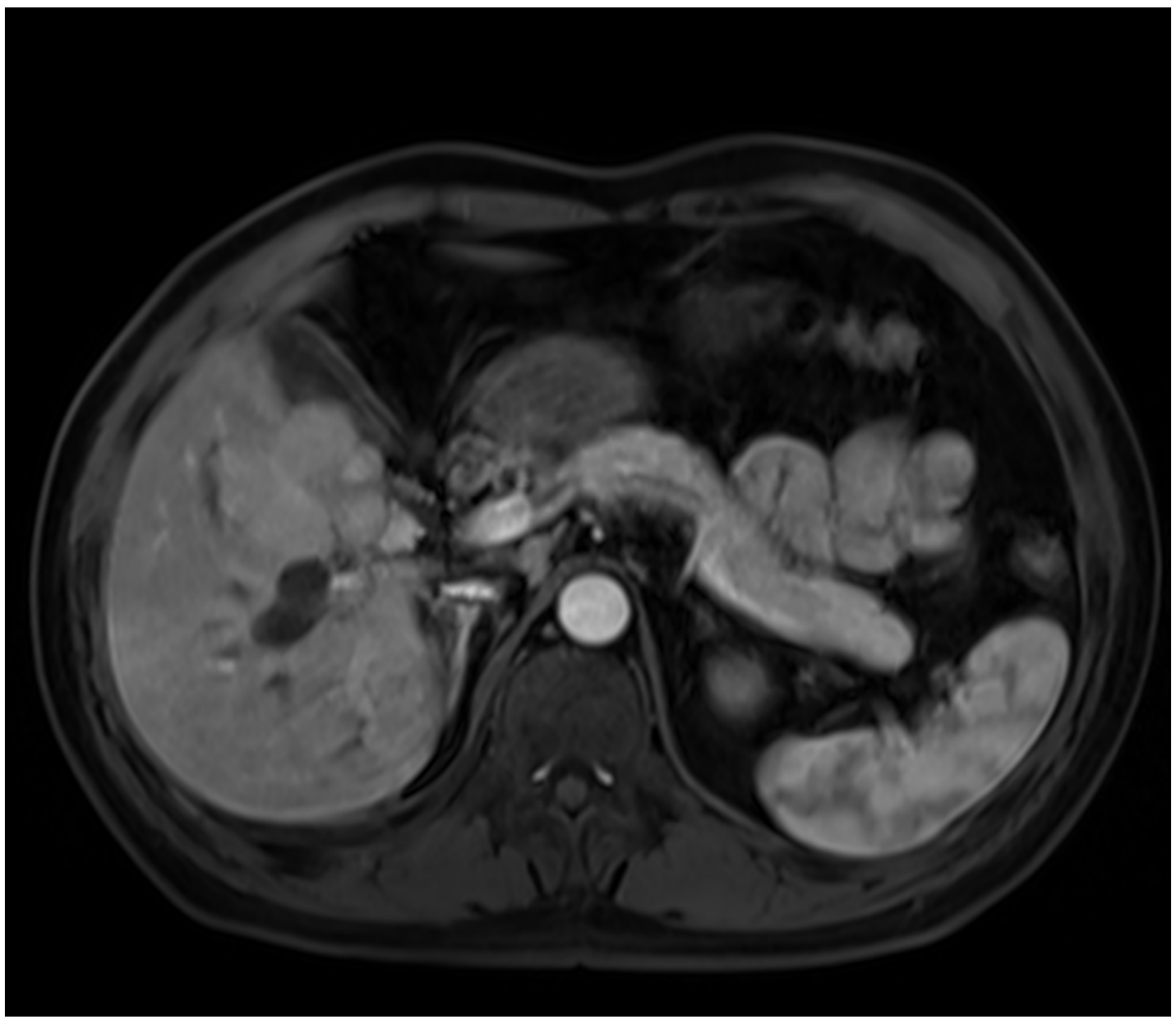

After treatment

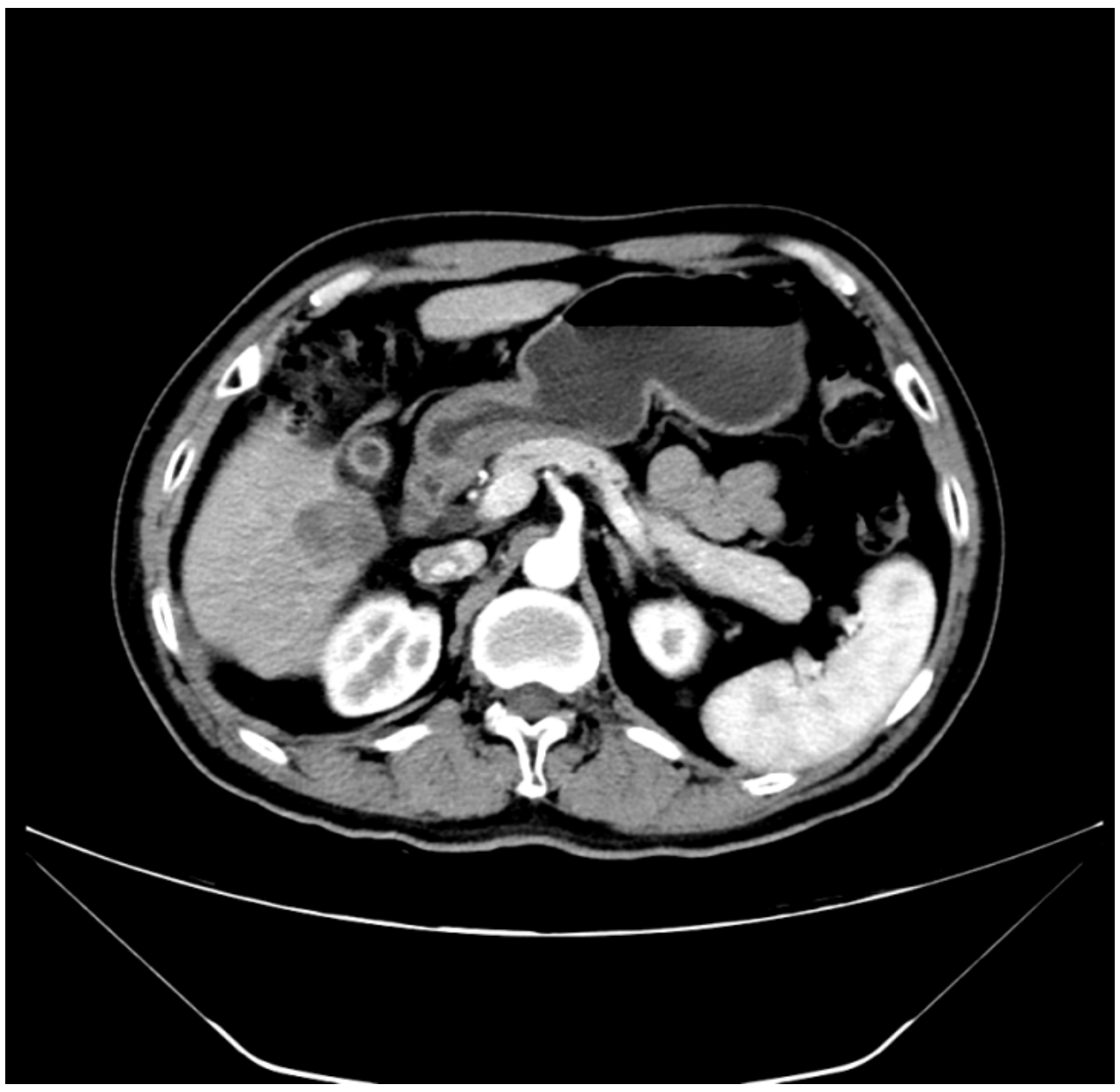

SoraHAIC Patient 03

Before treatment

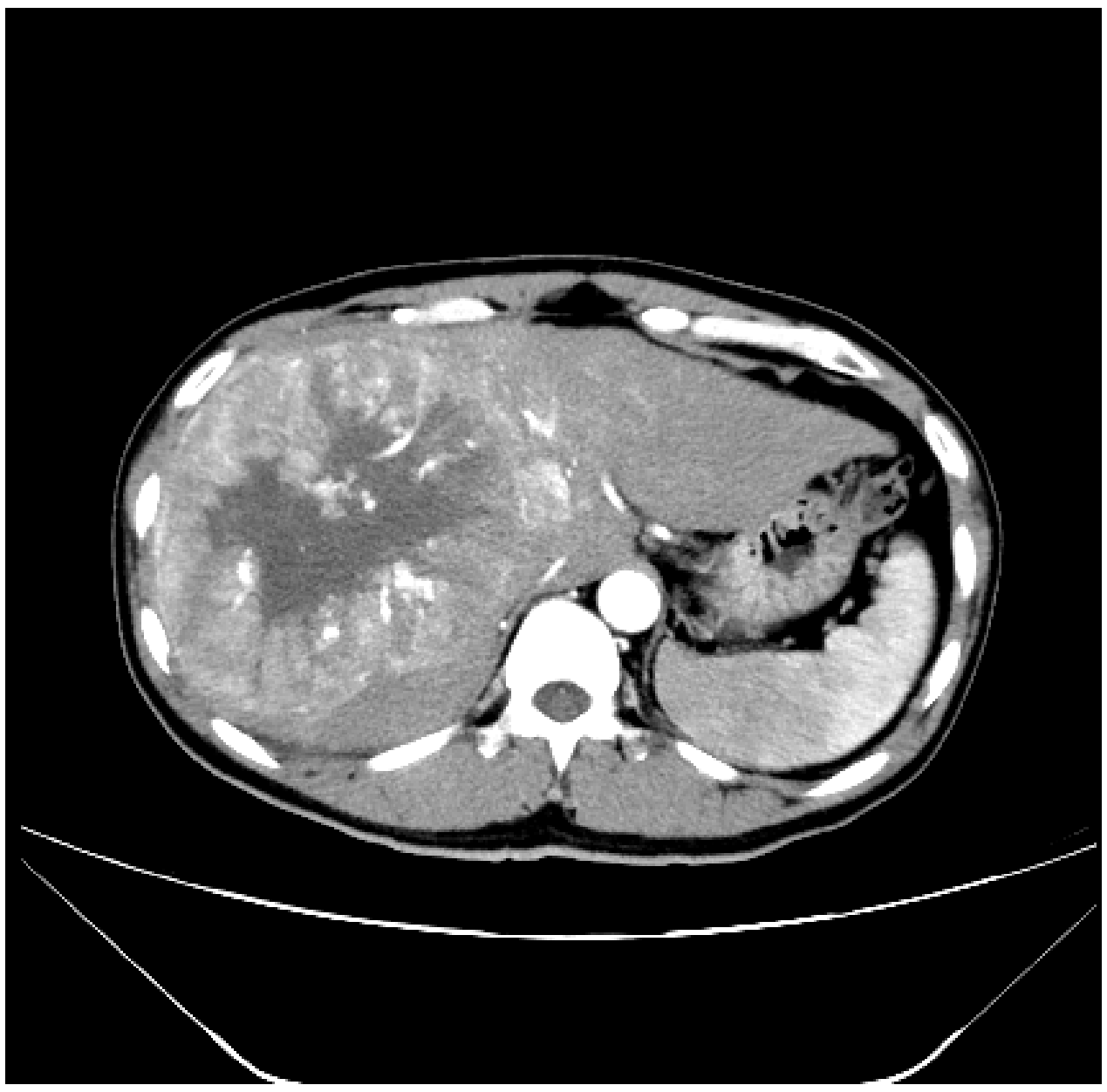

After treatment

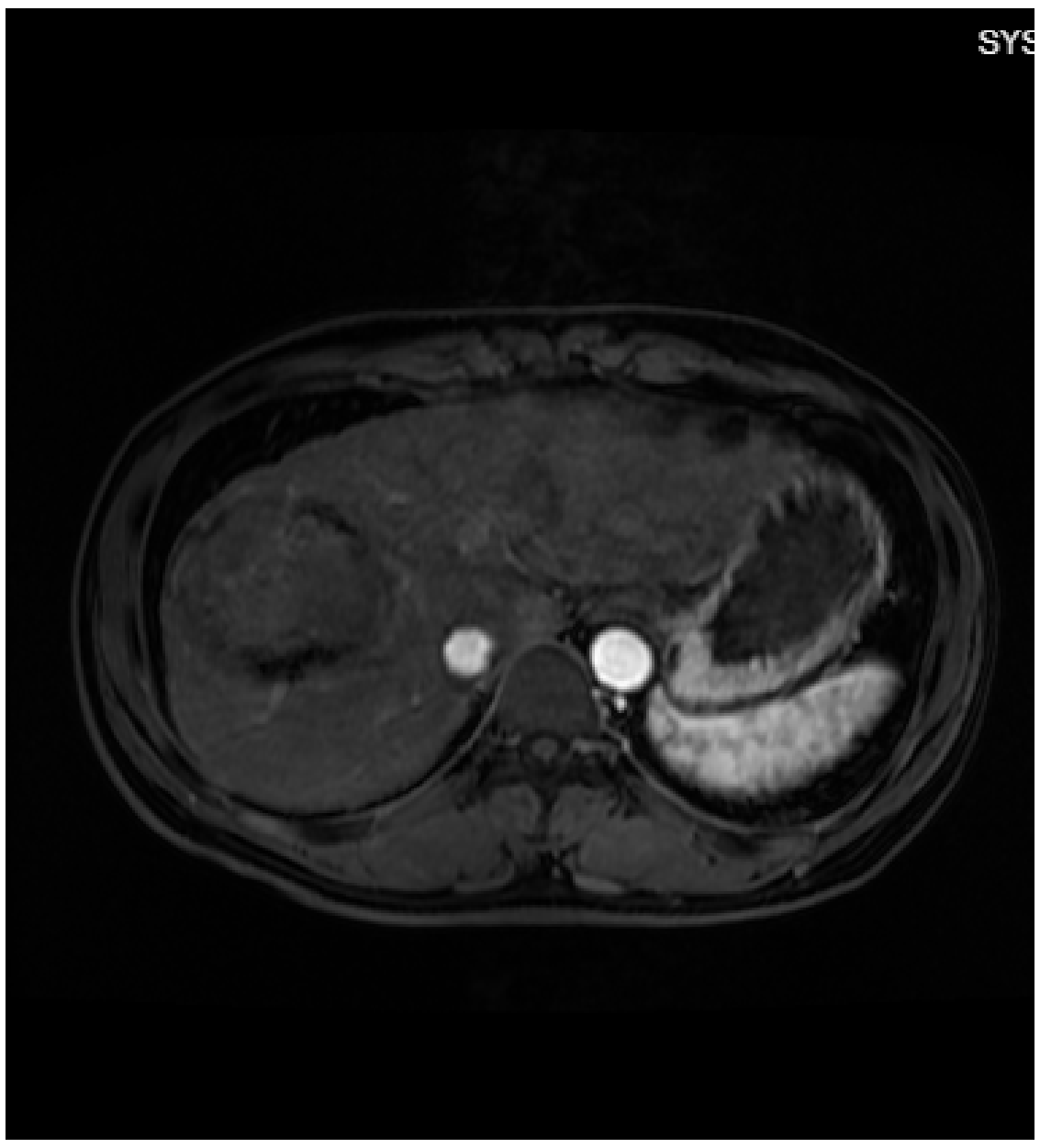

SoraHAIC Patient 04

Before treatment

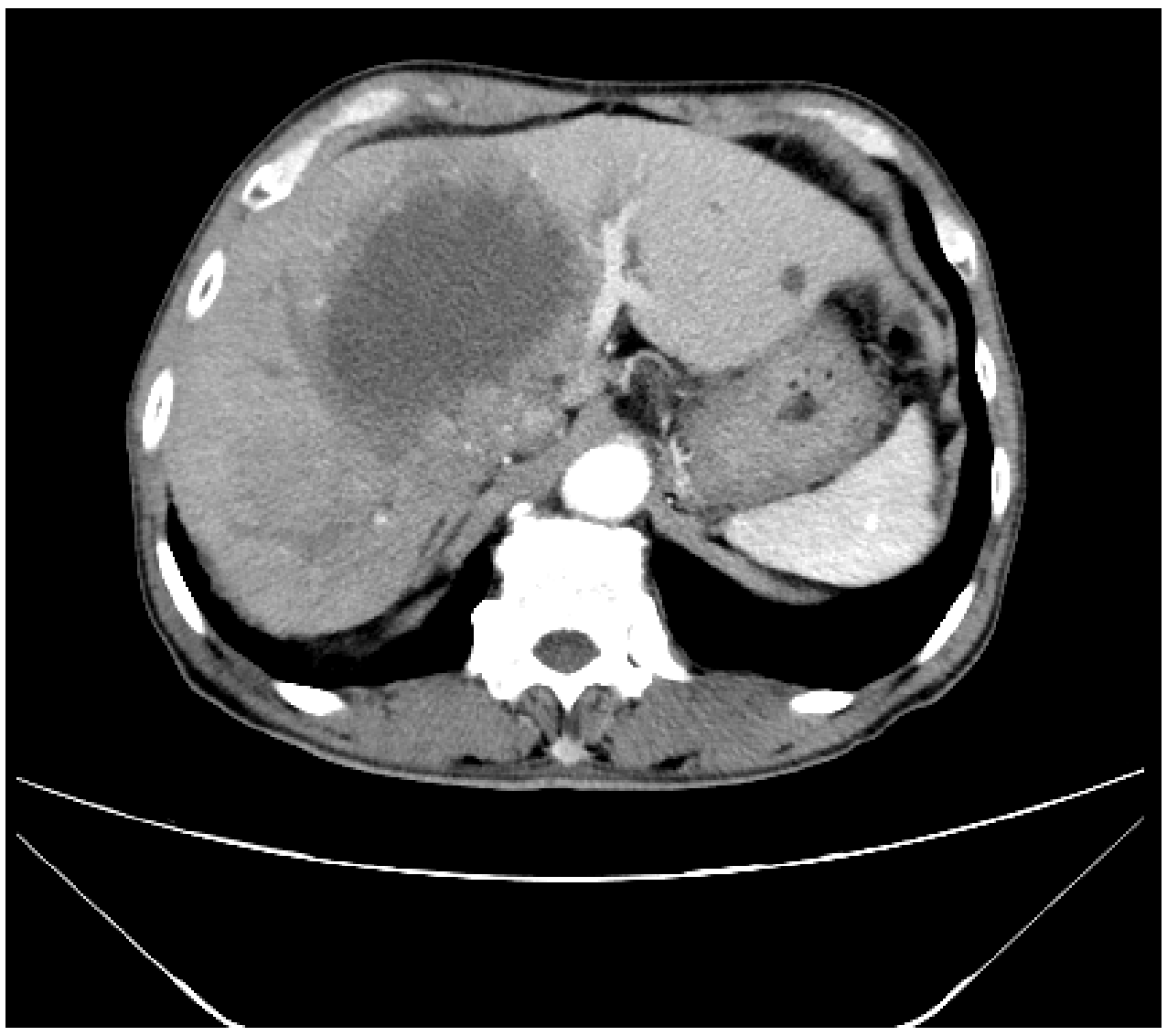

After treatment

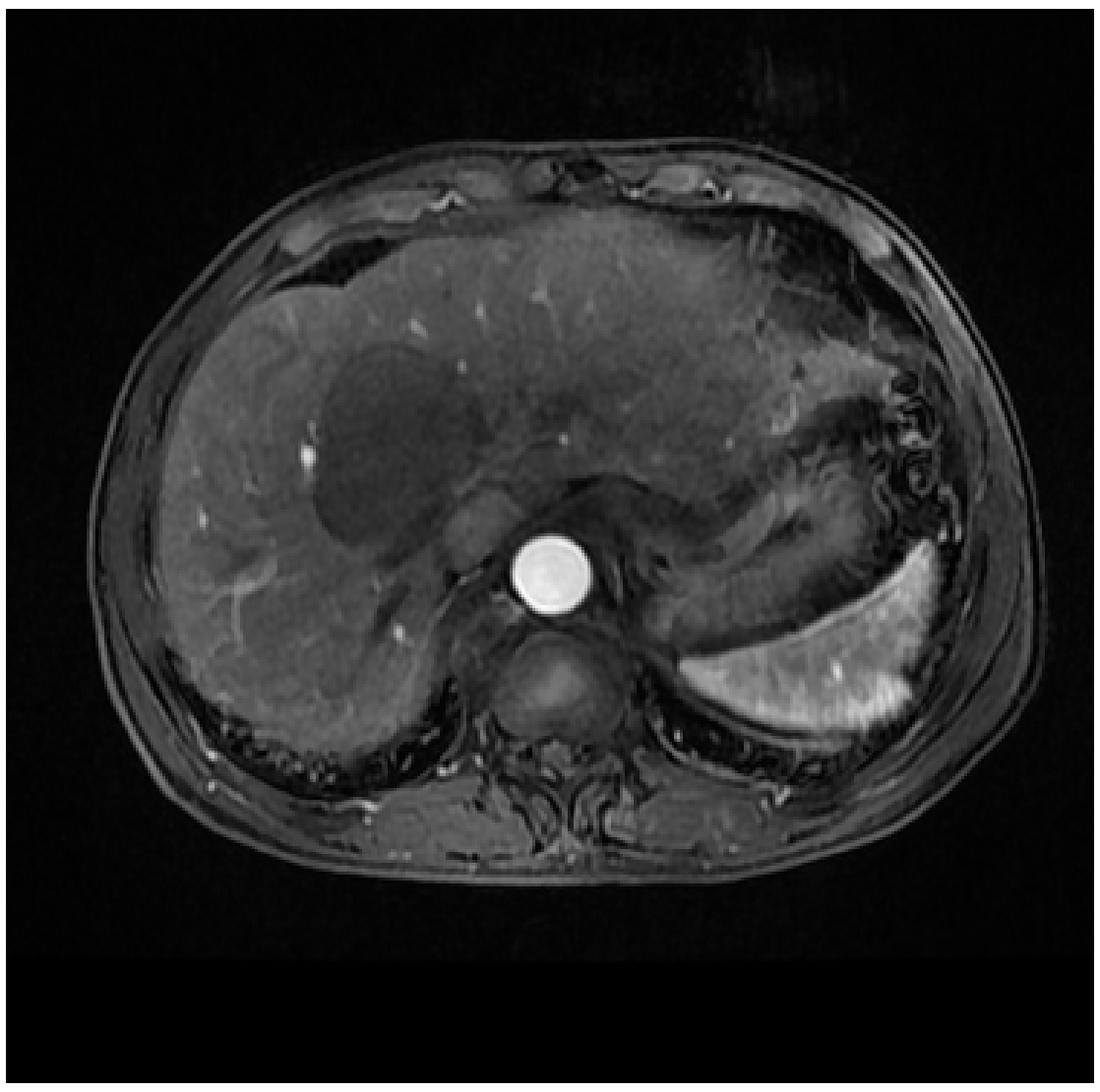

SoraHAIC Patient 05

Before treatment

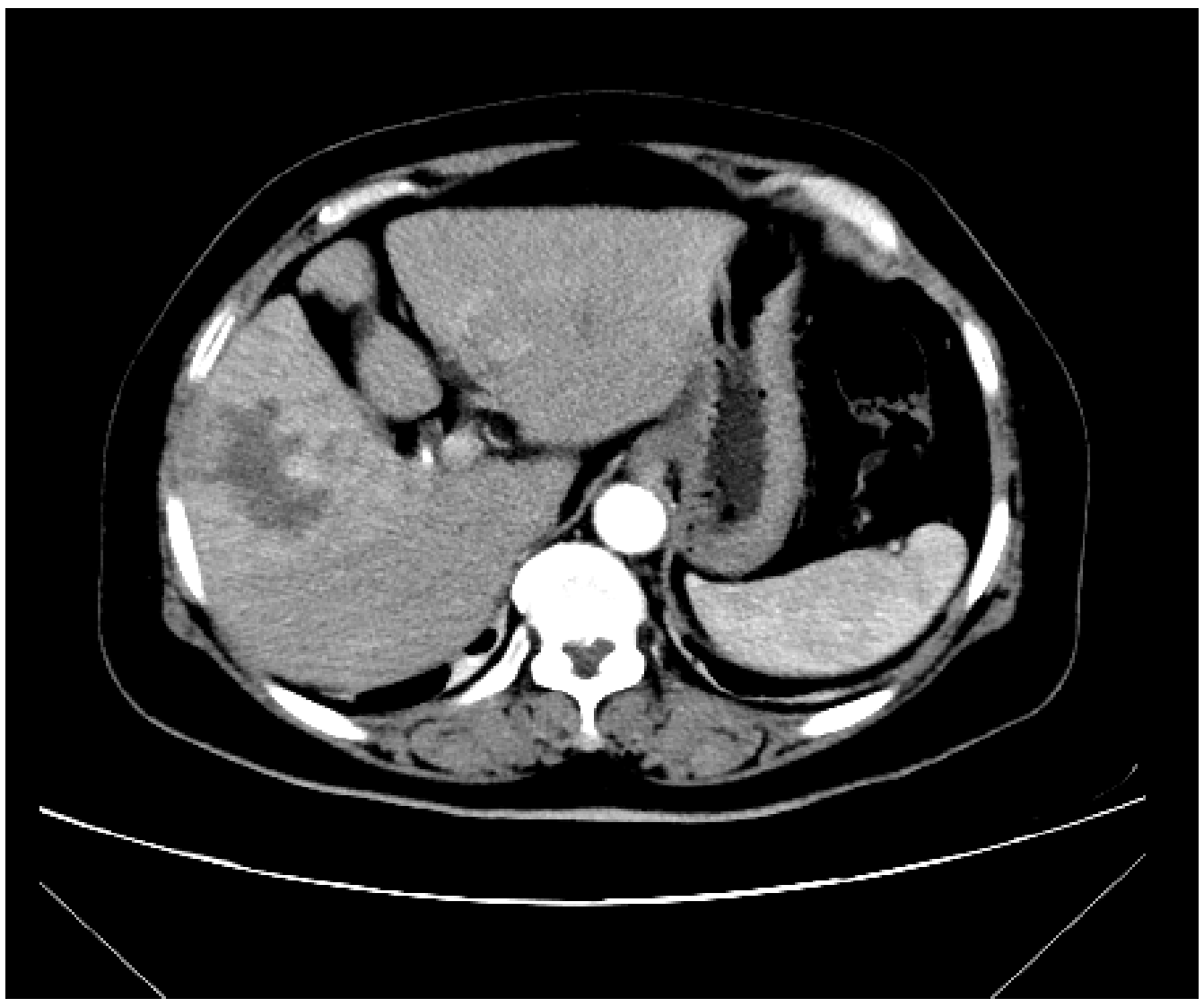

After treatment

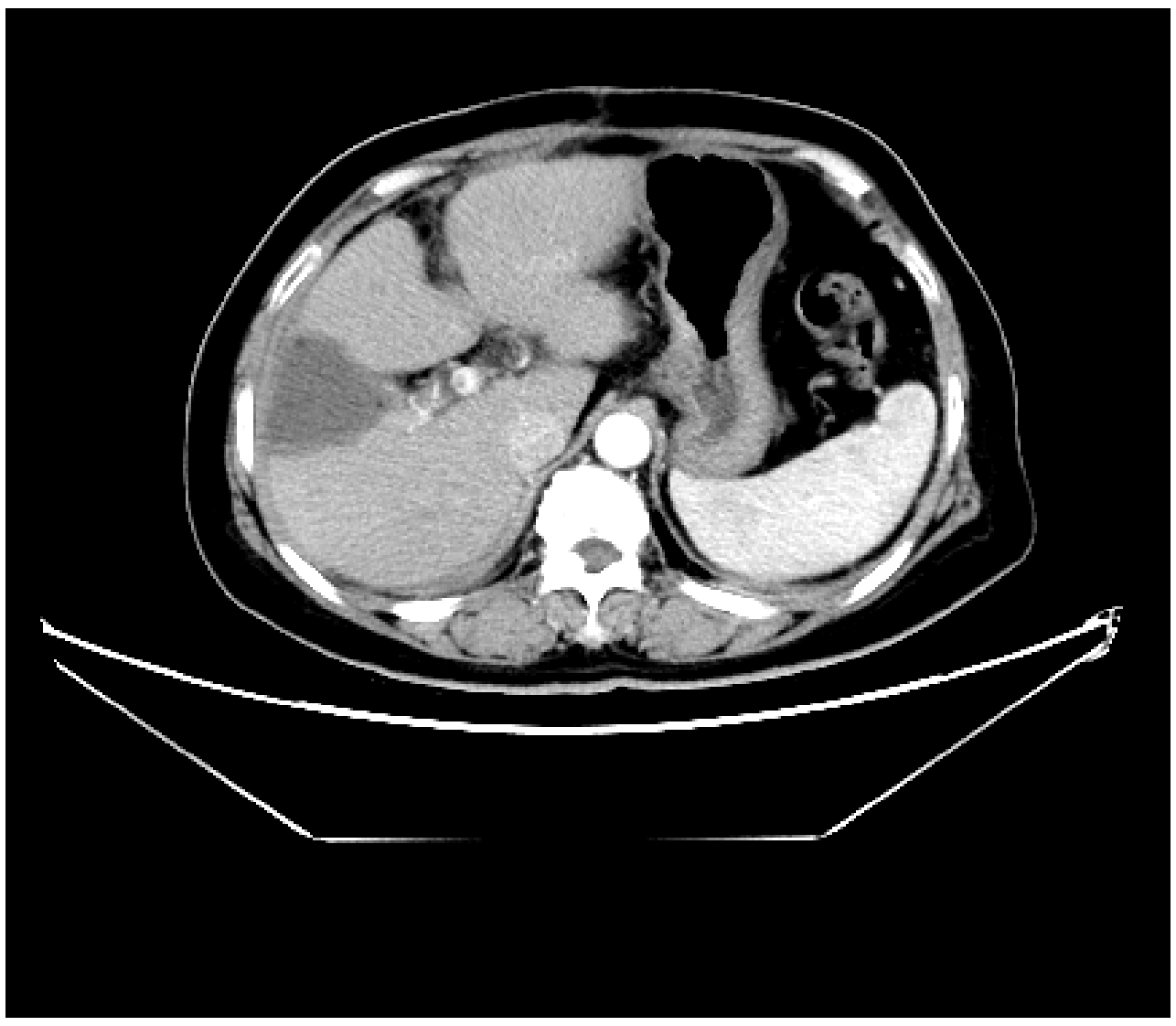

SoraTACE Patient 01

Before treatment

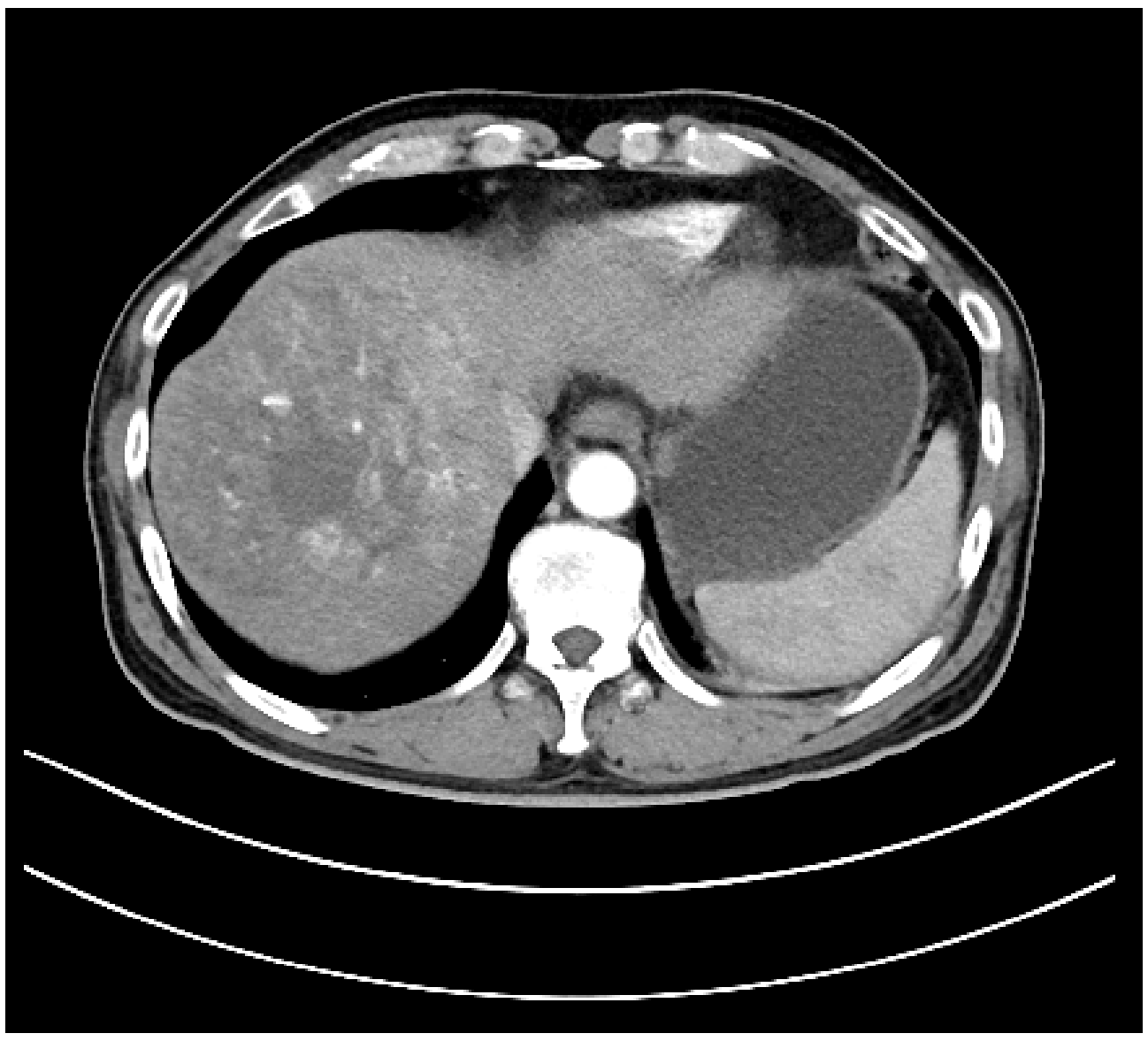

After treatment

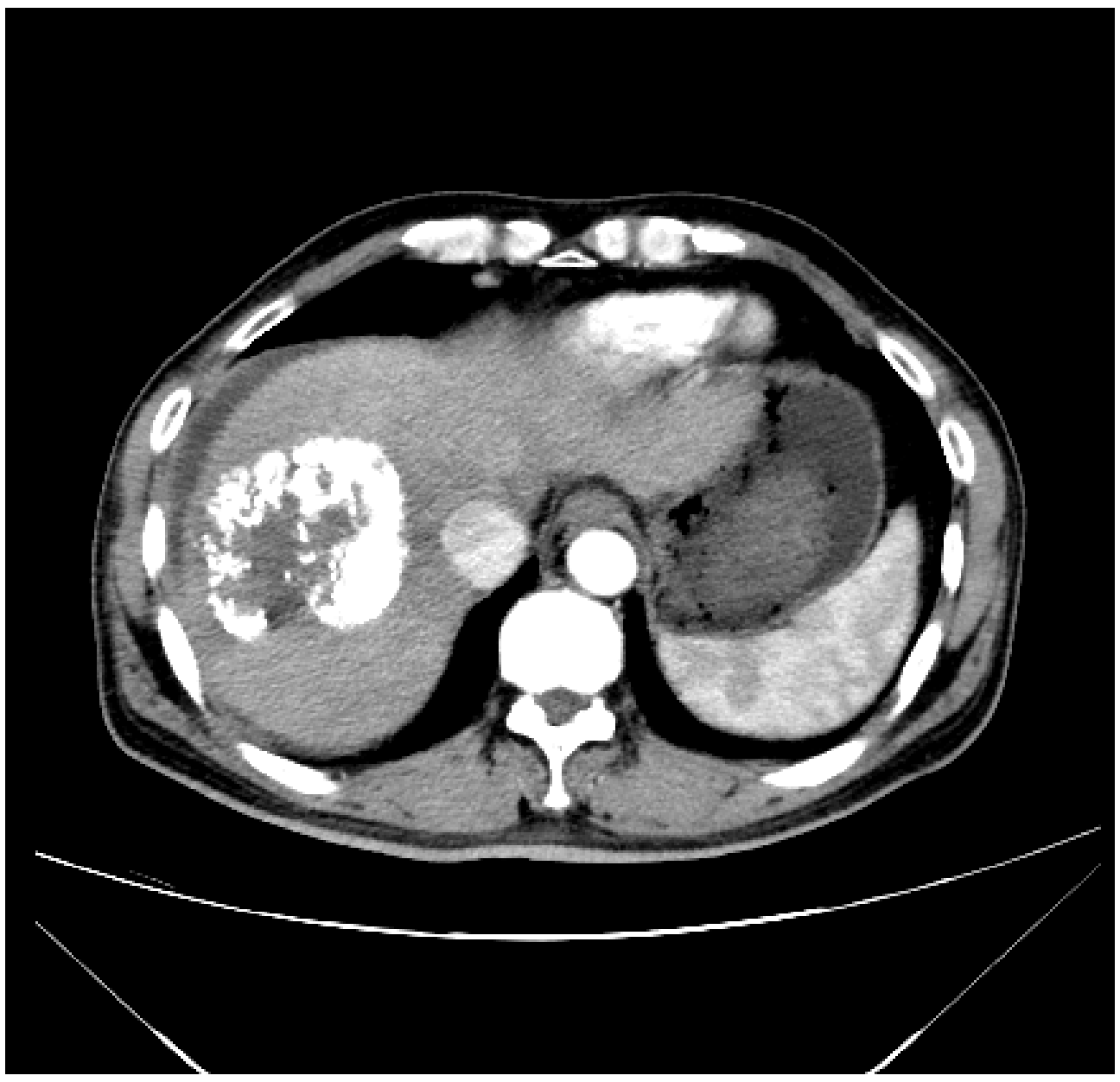

SoraTACE Patient 02

Before treatment

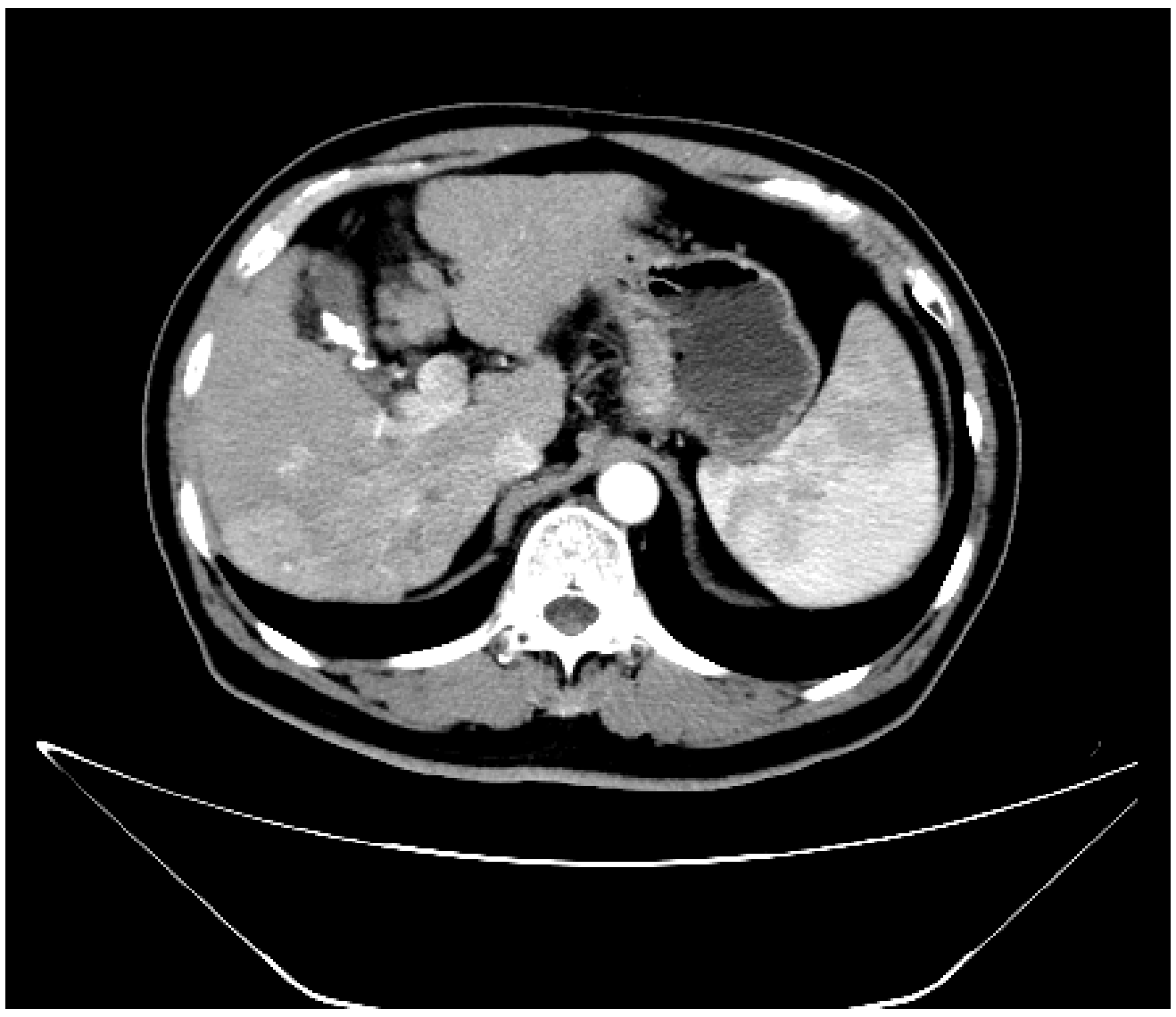

After treatment

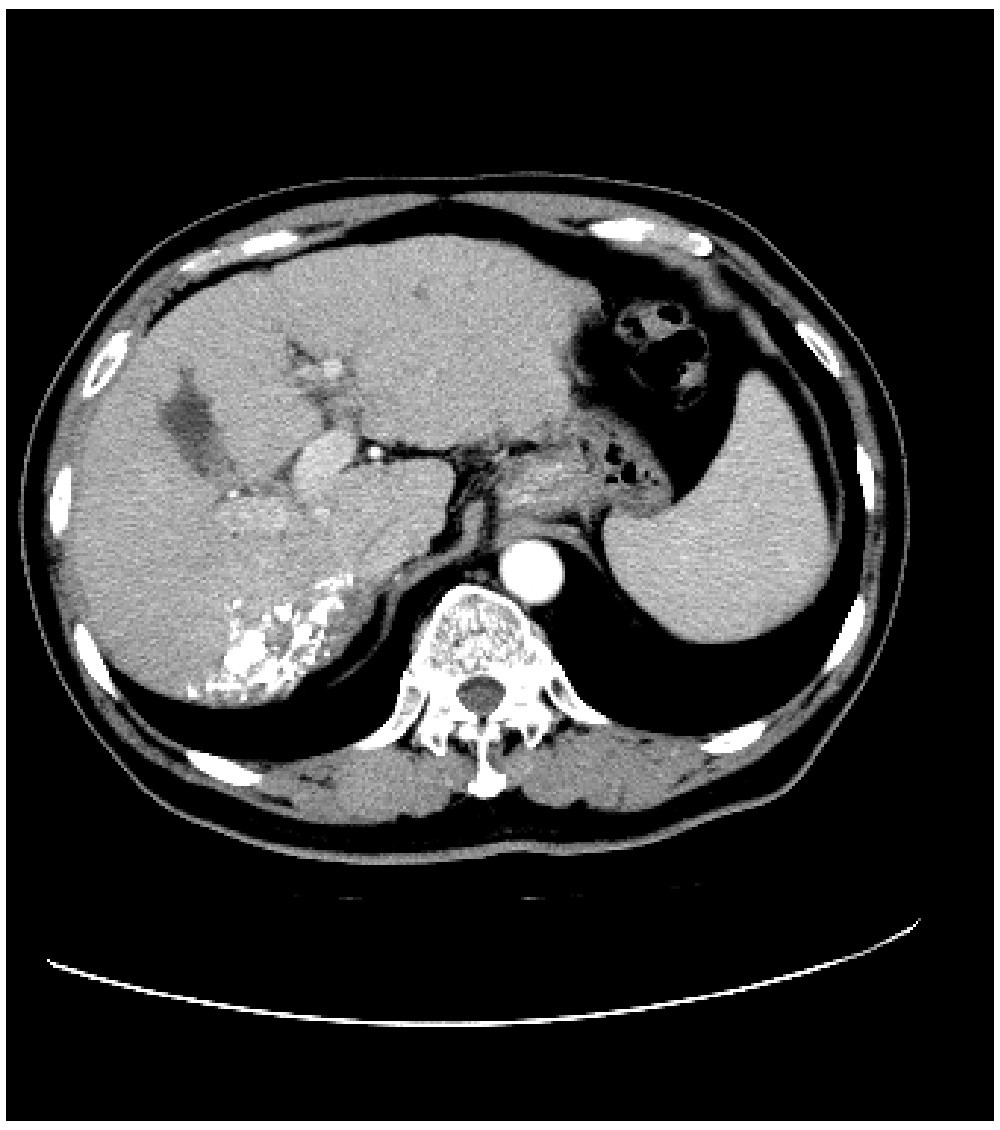

Supplement: Supplementary file 1 [file mmc1.pdf]
